# Decoding the Dynamics of Cultural Change: A Cultural Evolution Approach to the Psychology of Acculturation

**DOI:** 10.1177/10888683241258406

**Published:** 2024-07-26

**Authors:** Jonas R. Kunst, Alex Mesoudi

**Affiliations:** 1 University of Oslo, Norway; 2 University of Exeter, United Kingdom

**Keywords:** acculturation, conformity bias, cultural evolution, cultural change, immigration, majority, minority, payoff bias, vertical transmission

## Abstract

Although acculturation psychology is extensively studied in the social sciences, research progress has slowed due to overused methodologies and theories and emerging challenges to core conceptual tenets. Here, we seek to stimulate scientific inquiry into acculturation by integrating underutilized cultural evolutionary perspectives. We propose that cultural evolutionary mechanisms, such as (anti)conformity, prestige bias, payoff bias, and vertical transmission are instrumental in understanding when, why, and how minority- and majority-group members acculturate. The direction and potency of these mechanisms are proposed to be modulated by a combination of contextual and individual factors, resulting in acculturation strategies that at the population level form “cultural evolutionary equilibria.” These equilibria in turn have consequences for the long-term, population-level dynamics of cultural evolution. We outline how our integration of perspectives can allow researchers to model the dynamics of large-scale cultural change, increasing our understanding of the complex challenges faced by today’s diverse societies.

**Public Abstract:**

Acculturation describes the cultural and psychological changes resulting from intercultural contact. Here, we use concepts from “cultural evolution” to better understand the processes of acculturation. Cultural evolution researchers view cultural change as an evolutionary process, allowing them to borrow tools and methods from biology. Cultural evolutionary mechanisms such as conformity (copying the numerical majority), anti-conformity (copying the numerical minority), prestige bias (copying famous individuals), payoff bias (copying successful people), and vertical cultural transmission (copying your parents) can cause people to adopt elements from other cultures and/or conserve their cultural heritage. We explore how these transmission mechanisms might create distinct acculturation strategies, shaping cultural change and diversity over the long-term. This theoretical integration can pave the way for a more sophisticated understanding of the pervasive cultural shifts occurring in many ethnically diverse societies, notably by identifying conditions that empower minority-group members, often marginalized, to significantly influence the majority group and society.

Migration is an integral aspect of human history. From ancient human groups migrating across continents to modern population flows across borders, the pursuit of better opportunities, safety, and prosperity has driven the continuous flow of people across geographical boundaries. Recently, however, shifts toward globalization have drastically reshaped the pace and scale of migration. According to the International Organization for Migration, the number of international migrants has tripled to 281 million since 1970, constituting about 3.6% of the global population (International Organization for Migration [IOM], 2022). Consequently, social scientists are increasingly interested in how immigrants and citizens of immigrant-receiving societies respond to migration and how these responses affect wider society.

Within psychology, the cultural and psychological changes that result from intercultural contact are studied in the field of acculturation (Berry, 1980; Berry & Sam, 1997; Sam & Berry, 2016). It is well-established that acculturation can be optimally characterized as a multidimensional process (Ryder et al., 2000) where individuals simultaneously differ on various, relatively orthogonal dimensions of which two have received particular attention: the extent to which they retain their heritage cultures and the extent to which they adopt elements from other cultures. Large-scale investigations and meta-analyses suggest that most individuals follow the integration strategy (Berry et al., 2006a; Bierwiaczonek & Kunst, 2021)—they maintain aspects of their heritage culture while simultaneously adopting elements of the dominant majority culture.

Recently, however, the acculturation psychology field has plateaued in its development, with a stagnation in groundbreaking discoveries, a saturation of current methodologies, and research questioning its fundamental assumptions (Kunst, 2021). Crucially, recent meta-analyses cast doubt on the extent to which acculturation orientations predict immigrants’ well-being and socio-cultural functioning (Bierwiaczonek & Kunst, 2021). If improving well-being or social functioning is not the primary purpose of acculturation orientations and strategies, alternative explanations are needed for the disproportionate prevalence of certain acculturation strategies. Moreover, although acculturation is typically defined as a two-way process causing change in all cultural groups involved in intercultural contact, most research has ignored cultural change among majority-group members living in increasingly diverse societies (Kunst et al., 2021). Therefore, it is largely unknown whether the same processes that govern the acculturation of minority-group members also cause the acculturation of majority-group members.

In this context, our objective here is to invigorate the scientific study of acculturation through the integration of a cultural evolutionary perspective. The field of cultural evolution (Boyd & Richerson, 2005; Creanza et al., 2017a; Henrich, 2015; Mesoudi, 2011a) aims to explain cultural change and diversity within an evolutionary framework, going beyond the traditional and simplistic “evolution vs. culture” dichotomy common in the social sciences. Instead, cultural change is itself seen as an evolutionary process, one in which ideas, skills, attitudes, customs, norms, institutions, and other cultural traits are passed from person to person via social learning. This process forms a cultural inheritance system that acts in parallel to genetic inheritance. Where cultural traits vary in a population, often some variants are more likely to be passed on than others. This selective transmission might be due to the intrinsic features of traits (e.g., the efficiency of a tool or the catchiness of a tune), or qualities of the individuals bearing those traits (e.g., their prestige). Just as certain genes increase in frequency due to natural selection so too cultural variants increase in frequency due to these sources of cultural selection. Such cultural selection biases, as well as migration and innovation, shape cultural diversity across populations and drive cultural change over time.

Despite the high potential for using concepts and methods from cultural evolution to shed light on acculturation, only a handful of studies have attempted this to date and in a piecemeal way. Therefore, our aim here is to synthesize major theoretical perspectives from the two fields of acculturation psychology and cultural evolution. Specifically, our goal is to outline how social learning processes—including conformity, anti-conformity, payoff bias, prestige bias, and vertical cultural transmission—can elucidate the circumstances that lead to the adoption of cultural elements held by minority- and majority-group members as well as the preservation of their own cultural heritage. Furthermore, we discuss how acculturation strategies observed at the levels of individuals and populations, a frequent focus of study in acculturation psychology, may reflect “cultural evolutionary equilibria” due to the interplay of these processes with contextual and individual factors. Finally, we explore the long-term, society-level consequences of these strategies and equilibria, such as how they shape cultural diversity, drive or hinder cumulative cultural evolution, and affect society-level cooperation and conflict. We believe that integrating these perspectives provides novel insights into the forms, causes and consequences of acculturation that can inform future theoretical and empirical research and have societal implications.

It is crucial to delineate the boundaries of our review and clarify our focus on the strand labeled “cultural evolution.” This focus is not indicative of a disregard for other perspectives within the broader literature seeking to understand cultural change but rather a strategic decision to delve into the specific psychological processes and predictive mechanisms encapsulated within the cultural evolution framework. Thus, the particular cultural evolutionary mechanisms explored herein are not posited as the sole drivers of cultural change (see Varnum & Grossmann, 2017 for a review). For instance, the selective retention of information within groups and the modeling of how social influence can lead to attitudinal and behavioral shifts may help understand cultural dynamics (Harton et al., 1998; Latané, 1996; MacCoun, 2012). Likewise, evolutionary mechanisms have been applied to explain group decision-making processes (Tindale & Kameda, 2017), which can exert cultural influences over time. A comprehensive review of all theories of cultural change and their relation to acculturation is beyond the scope of a single review; hence, our exclusive focus on cultural evolution, which over the last 50 years has amassed an extensive body of formal theoretical and empirical work that perhaps uniquely straddles multiple academic disciplines, from anthropology and archeology to linguistics and psychology.

## Author Positionality Statement

The authorship of this article represents a convergence of distinct experiences and academic traditions. We both hail from culturally diverse families and were raised in Western societies. The first author is of Caucasian descent, with family members from Northern, Middle, and Eastern Europe and has personal experiences of migration within the Western hemisphere as a child and adult. The second author carries a mixed heritage of Western European and North African cultures and has lived and worked in Western European and North American countries. Moreover, we both have partners from minority cultures within our societies. Although these experiences provide personal perspectives on the acculturation process, we acknowledge the limits of these viewpoints, as we lack firsthand understanding of the challenges faced by migrants from disadvantaged backgrounds. While our familial and personal contexts have surely stoked our interest in topics related to diversity and immigration, they may also potentially predispose us toward a more positive viewpoint on these issues. We acknowledge the risk of allowing our personal experiences to unduly influence our theorizing.

Our academic backgrounds also bring unique biases and insights. One of us was trained in traditional social and cultural psychology, while the other in the evolutionary human sciences, holding academic positions in both (biological) anthropology and biology departments. The discourse between these fields is often characterized by critique and bias. Social and cultural psychologists often criticize evolutionary approaches to human behavior for their perceived biological determinism and neglect of culture, while evolutionary human scientists often criticize social psychology’s alleged neglect of our species’ evolutionary origins and evolutionary influences on the mind. Despite these differences, we view our disparate backgrounds and perspectives as an opportunity to provide a balanced and integrative review. While we have endeavored to be as impartial as possible, we recognize that our preferred theoretical frameworks and personal experiences shaped the outcome of our collaboration.

## The Need to Revisit the Principles of Acculturation Psychology in a Rapidly Changing World

Before detailing how cultural evolution theory can inform acculturation research, it is worth highlighting how timely such an integration is, and briefly reviewing the current state of acculturation psychology research. As noted above, migration is a defining feature of the human species, from our African origins and initial expansion across the planet (Bergström et al., 2021), to more recent mass migration from the old to the new world (Hatton & Williamson, 1998). Even in this context, however, the current rate and acceleration of human migration is unparalleled in history. Between 1990 and 2022 alone, the number of international migrants increased from 49.6 million to 86.7 million in Europe, from 48.2 million to 85.6 million in Asia, from 27.6 million to 58.7 million in Northern America, from 15.7 million to 25.4 million in Africa, from 7.14 million to 14.8 million in Latin America and the Caribbean, and from 4.73 million to 9.38 million in Oceania (IOM, 2022). Moreover, these figures only account for individuals who have crossed national borders during their lifetime, not the increasing number of individuals with migration backgrounds (e.g., those born to immigrant parents or grandparents). Consequently, the transformation of traditionally homogeneous nations into ethnically diverse societies becomes even more stark. For example, in contemporary Germany, one in four individuals possesses a migration background (Destatis, 2023). In France, nearly one-third of all newborns have at least one parent born abroad (Insee, 2023), while in the United Kingdom, more than a quarter of all children have at least one foreign-born parent (The Migration Observatory, 2022).

It is important to recognize the heterogeneity among groups within the broader migrant population. These groups can be classified according to their migration motivations, duration of residency, and voluntariness of intercultural engagement (Berry & Sam, 2016). Three principal categories encapsulate a significant portion of migrants: sojourners, immigrants, and refugees. Each category occupies distinct social positions, which profoundly affect their acculturation processes and adaptation requirements (Kuo, 2014). Sojourners are individuals who choose to relocate to a new country temporarily, typically for education or short-term employment. The acculturation outcomes for sojourners may not be as pivotal for their life paths, albeit with certain nuances. Expatriates, possessing coveted expertise or professional qualifications, find themselves in more favorable negotiation positions compared with other migrants, potentially reducing the pressures to conform or engage with different groups (Safdar & Berno, 2016). In addition, expatriates often undergo intercultural training, preparing them for the acculturation process; however, the limited nature of their stays constrains the scope and impact of intercultural interactions. Another significant subset of sojourners includes international students who, driven by the globalization of higher education, increasingly pursue studies abroad, whether for short-term exchanges or entire degree programs. Various factors, such as language barriers, discrimination, and financial difficulties, may impede their ability to establish positive intercultural relationships with members of the majority group (Safdar & Berno, 2016).

Conversely, immigrants typically move with the primary and voluntary intent of seeking employment, family reunification, or romantic partnerships. Given their longer-term aspirations in the host society, immigrants are more likely to experience the necessity or find value in adapting to the dominant culture and engaging with other groups. However, immigrants from lower-status groups may encounter greater stigma and a less hospitable reception from the host society (Montreuil & Bourhis, 2001, 2004). Furthermore, their socioeconomic status and social mobility often dictate their residential choices (Iceland & Wilkes, 2006). For example, those with lower socioeconomic status may face segregation, limiting their opportunities for interaction with the majority population, especially in the absence of social mobility.

Finally, refugees represent the most vulnerable category, forced to flee their home countries due to persecution, conflict, or violence, in search of asylum or protection abroad. Refugees face considerable obstacles, including significant legal uncertainties and unwelcoming host environments (Dona & Young, 2016). They are often subject to stringent and sometimes adversarial immigration policies that profoundly influence their acculturation experiences. In addition, the isolation resulting from placement in remote detention centers severely restricts refugees’ opportunities for interaction with the majority community. When such interactions occur, they are frequently met with hostility, adversely affecting their well-being (da Silva Rebelo et al., 2018). The trauma associated with war and other adverse experiences further complicates their acculturation processes (Phillimore, 2011).

This heterogeneity combined with the aforementioned sharp increase in migration rates underscores the need to understand the acculturation of individuals who move from one cultural context to another, reflecting the recent surge in acculturation-related studies (Kunst, 2021). Most of this research builds on the influential model of acculturation formulated by Berry (1980, 1997). Initially, this model assumed that immigrants and minority-group members grapple with two fundamental issues: the degree to which they preserve their heritage culture and the degree to which they seek interaction with members of other groups. Subsequently, Bourhis et al. (1997) emphasized that immigrants and minority-group members not only seek interaction with but also adopt the culture of other groups, particularly the majority society. Berry and colleagues later themselves adopted this more encompassing approach (Berry et al., 2006a, 2006b).

A key element of Berry’s current acculturation model posits that immigrants can both maintain their own culture and adopt the mainstream culture in various ways, which broadly culminates in four acculturation strategies (Berry, 1997): integration (i.e., maintaining one’s original cultural identity while also adopting elements of other cultures), assimilation (i.e., adopting other cultures and relinquishing one’s heritage culture), separation (i.e., maintaining one’s heritage culture and rejecting other cultures), and marginalization (i.e., rejecting both one’s heritage and other cultures). A fundamental assumption of acculturation theory is that different strategies yield distinct consequences for immigrants’ well-being and sociocultural functioning (Berry & Sam, 1997). The “integration hypothesis” (Berry, 2013) assumes that integration fosters the most favorable psychological and sociocultural outcomes. This causal link is widely assumed to explain why most immigrants appear to adopt the integration strategy (Berry et al., 2006b) but has recently been questioned. A re-analysis of a highly influential meta-analysis revealed that the correlation between integration and psychological well-being and sociocultural functioning is considerably smaller than previously assumed, whereas a new longitudinal meta-analysis failed to identify any consistent temporal effect (Bierwiaczonek & Kunst, 2021; Kunst, 2021). Similarly, a recent longitudinal study found no statistically significant influence of acculturation strategies on social support, or vice versa (Zagefka et al., 2023).

If integration has minimal consequences for the well-being and sociocultural functioning of immigrants and minority-group members, what other factors might explain the predominance of the integration strategy as well as the existence of other strategies at lower frequencies? Berry et al. (2002) themselves suggested that cultural transmission processes, typically examined within the field of cultural evolution, might explain the development of individuals’ cultural self-concepts and acculturation. Nonetheless, a comprehensive theoretical account of these dynamics, along with their individual, group, and contextual moderators, remains absent. Most critically, acculturation models have predominantly concentrated on acculturation as an individual-level phenomenon, rarely extrapolating it to the population level or considering its evolutionary basis or consequences. Thus, we suggest that acculturation theory currently is not adequately equipped to explain the large-scale patterns of cultural change and diversity in the world today.

Furthermore, although Berry’s original model could potentially be applied to the acculturation of both majority and minority groups, research focusing on majority groups has predominantly concentrated on the acculturation they anticipate and expect from immigrants, what predicts these expectations, and how these expectations are perceived by migrants (Bourhis et al., 1997; Horenczyk et al., 2013; Navas et al., 2005; Piontkowski et al., 2002). While these are important extensions of Berry’s original framework, they do not sufficiently consider how majority groups, just like minority groups, undergo acculturation as a consequence of contact with other cultural groups. The aforementioned surge in global immigration reinforces the need for acculturation research and theory to explore the acculturation of majority-group members and its consequences for increasingly ethnically diverse societies. Given the selective settling of migrants in specific geographic regions, many countries, cities, and neighborhoods now contain such a degree of diversity that the erstwhile ethnic majority group now constitutes a numerical minority in comparison to all other ethnic groups combined (Gest, 2022). Recent statistics from countries such as the United States vividly demonstrate these changes. Between 2000 and 2018, the proportion of the White population declined to below 50% in 109 U.S. counties (Pew Research Center, 2019). Importantly, these demographic shifts are not confined to settler societies but are observable in many other parts of the world, including a variety of large cities (Crul, 2015). In these emerging “majority-minority” contexts, the need to comprehend the acculturation of majority groups becomes increasingly important.

Recent research has attempted to modify Berry’s model to incorporate the acculturation of majority groups, which typically wield more power and influence than immigrant groups and therefore are in a fundamentally different acculturating position (Kunst et al., 2021). However, while this research has identified acculturation strategies and their social and personality psychological correlates, it also fails to elucidate why these patterns emerge and how they may lead to large-scale cultural change. As concepts such as polyculturalism suggest, cultural groups do influence each other, and the direction of influence is not solely unidimensional but dynamic, multifaceted, and multileveled (Rosenthal & Levy, 2012). Some members of majority groups appear to adopt hybrid identities in response to this complex exposure to various cultural influences (Chen et al., 2016).

**Figure 1.**
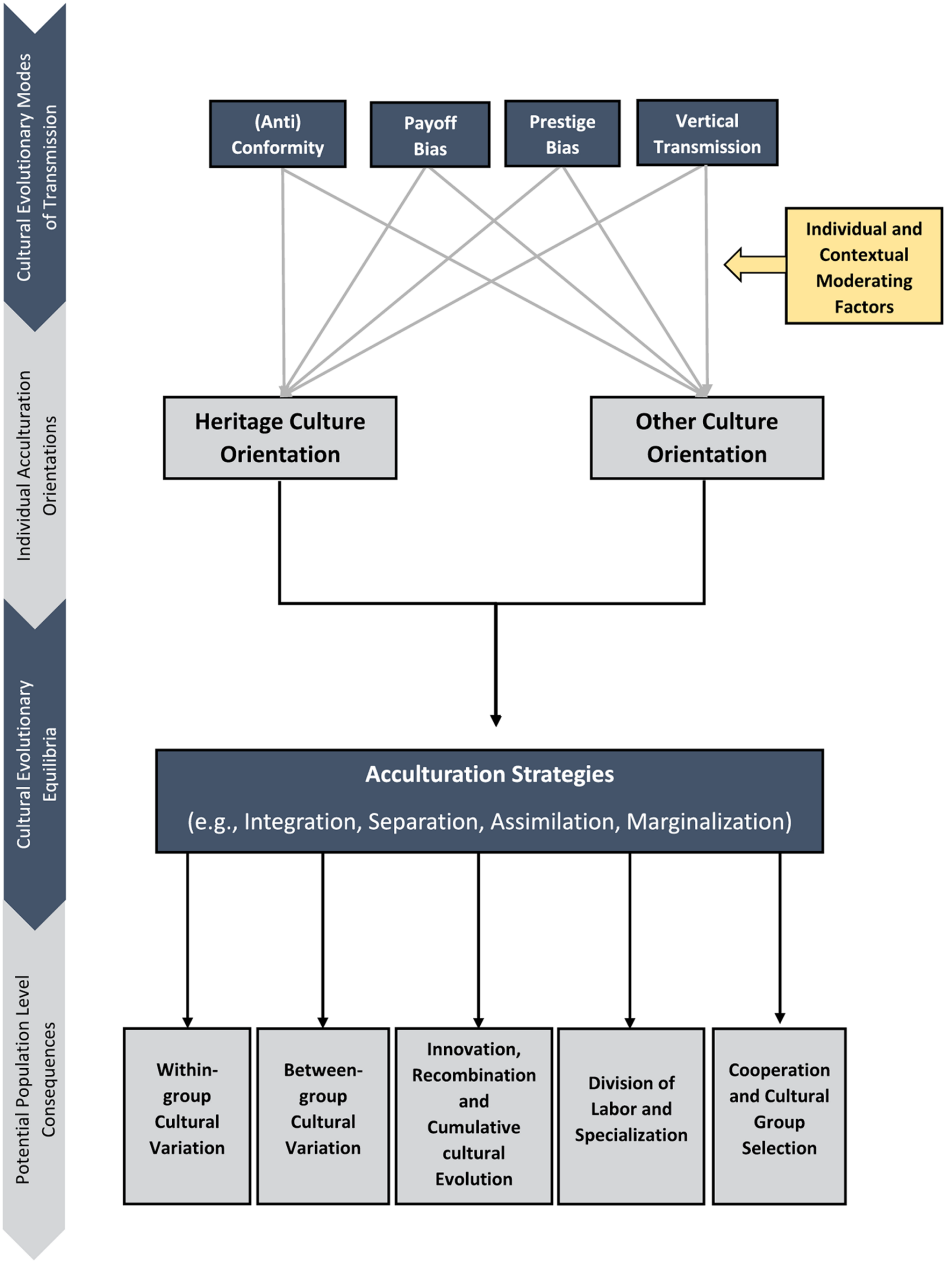
The Proposed Cultural Evolutionary Model of Acculturation.

Against this backdrop, the present paper proposes a fundamentally distinct approach to understanding the acculturation of both minority and majority groups (see Figure 1). At its core, it delineates how a suite of robust cultural evolutionary processes of cultural transmission can lead to acculturation orientations and strategies that are primarily treated as endpoints or “cultural equilibria” with population-level consequences that we detail subsequently. Our approach sketches out the individual, group, and contextual factors implicated in the acculturation process, based on prior research, that may navigate acculturating individuals away from or toward certain cultural streams. Inherent in this approach is its potential to bridge the gap between environmental cues and characteristics, individual acculturation, and population-level cultural change, thereby giving rise to various novel hypotheses that are testable through a cultural evolutionary framework.

**Box 1. Conformity and its population-level consequences**

To illustrate both the precise meaning of conformity in cultural evolution and the value of formal mathematical modeling, we here step the reader through a formal model of conformity (see also Henrich & Boyd, 1998; Mesoudi, 2021; Nakahashi et al., 2012). Imagine a population of individuals each of whom possess one of two cultural traits, A and B. These might be chopstick versus knife-and-fork use as in the main text, individualism versus collectivism, speaking German versus Turkish, and so on. For simplicity, these are discrete rather than continuous, and individuals cannot possess both. Obviously, these assumptions are unrealistic, but they serve our purposes in illustrating the effects of conformity here. In each timestep (e.g., a month, year, generation, or any other regular time interval), every individual surveys the frequency of trait A across the entire population, a proportion from 0 to 1 which we denote *x*. The frequency of B is therefore 1−*x*. When *x* = 1 everyone has trait A, when *x* = 0 everyone has trait B. Individuals then adopt trait A with probability equal to the frequency of trait A, modified by a conformity parameter *f*, as per Equation 1:

(1) $$P(A)=\frac{x^{f}}{x^{f}+{\left(1-x\right)}^{f}}$$

Here the probability of adopting trait A, *P(A)*, is equal to the frequency of trait A raised to the power *f*, divided by the sum of the frequencies of A and B each raised to the power *f*. When *f* = 1, the probability of A is exactly equal to the current frequency of A. This is unbiased, non-conformist social learning. As *f* increases, copying becomes more conformist, as shown in Table 1.

**Table 1.** Frequencies of Adoption for Conformist Social Learning.

		Frequency of trait A in population (*x*)					
		0	0.2	0.4	0.6	0.8	1
Probability of adopting trait A, *P*(A), assuming:	Unbiased, random copying (*f* = 1)	0	0.2	0.4	0.6	0.8	1
	Moderate conformity (*f* = 2)	0	0.06	0.31	0.69	0.94	1
	Strong conformity (*f* = 10)	0	0	0.02	0.98	1	1

**Figure 2.**
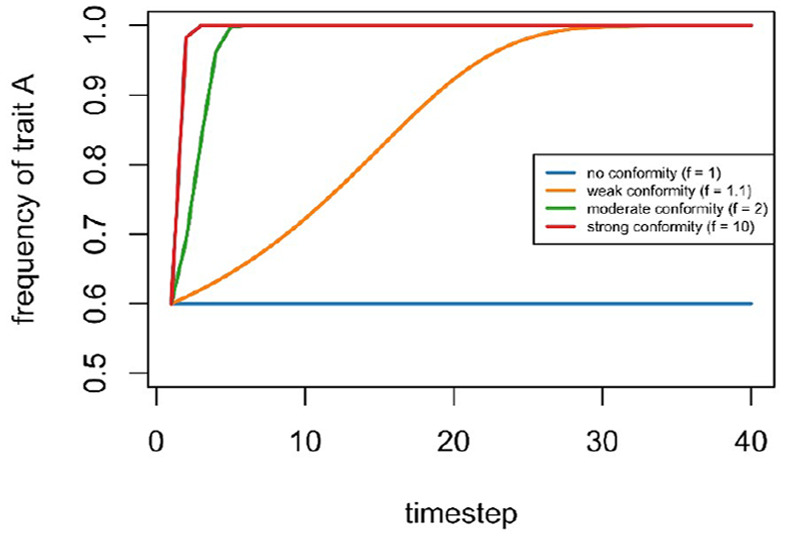
Visualizing the Frequency of an Initially More Common Trait Over Time at Different Levels of Conformity

Table 1 shows that with moderately strong conformity (*f* = 2), trait A is more likely to be adopted when it is more common than trait B (*x* > 0.5), and less likely to be adopted when it is less common (*x* < 0.5). This is what is meant by *disproportionate*: when A is common (e.g., *x* = 0.6), then even with unbiased copying A is more likely to be adopted, *P(A)* = 0.6, which would likely count as conformity according to traditional social psychological definitions; yet conformity here only occurs when this probability is greater than the baseline frequency, *P(A)* > 0.6. This difference might seem trivial, but unbiased copying and conformity in this sense have dramatically different population-level consequences. Figure 2 shows the long-term dynamics of *x* assuming different values of *f.* While unbiased copying does not change the frequency of trait A, conformity does, and even when weak (e.g., *f* =1.1) drives the initially most-common trait to fixation (i.e., to *x* = 1). This stark difference has important implications for cultural change and diversity (Mesoudi, 2018). With conformist social learning, we would expect majority norms to be maintained even if immigrants slightly change trait frequencies. For example, even if immigrants bring trait B into a population of mostly As, conformity will push the frequency of A back up to 1 unless immigration is so strong as to make A less common than B. If different groups have different majority norms, then conformity can maintain these between-group differences. With unbiased transmission, however, the decreased frequency of A will remain decreased, and with constant immigration, B may eventually go to fixation instead, or different groups will become identical mixes of A and B (Mesoudi, 2018). Without formal models, it is hard if not impossible for human minds to detect or predict these dynamics.

Note that two factors are varying in Table 1: the strength of conformity (*f*) and the frequency of the traits in the population (*x*). The former (*f*) might be seen as a psychological property of individuals, albeit one that in reality might vary across individuals, contexts, and societies, and respond to societal pressure, as discussed in the main text. The latter (*x*) might be seen as a demographic property of the population. In the model, the population is an undifferentiated mass of individuals each of whom is influenced by every other individual. In reality, different individuals might have different sets of individuals (e.g., their local neighborhood) from whom they derive their own personal *x*. This already suggests ways in which this simple model can be extended, such as by allowing *f* to vary across individuals, or explicitly modeling spatially segregated neighborhoods or social networks. Hence, simple models can ratchet up to fit different scenarios and phenomena, but without becoming too complex to be understood.

## Clarification of Terminology

Before exploring the potential role of cultural evolution in advancing our understanding of acculturation, it is critical to clarify terminology. First, there is typically confusion across fields regarding the term “evolution,” particularly in the context of cultural evolution and human behavior. We define “evolution” as any process that comprises variation, inheritance, and selection (Mesoudi et al., 2004). Genetic evolution comprises genetic variation, genetic inheritance, and natural selection. Cultural evolution comprises cultural variation, cultural inheritance (aka cultural transmission or social learning), and cultural selection. These two processes act in parallel and are partially independent; indeed, the field of gene-culture coevolution examines explicitly how genetic and cultural evolution interact. The key point is that when we discuss cultural evolutionary mechanisms or equilibria, there is no requirement that these involve genetic change or genetic differences between people. Moreover, in our review of cultural evolutionary processes, we will cover both individual-level learning biases (e.g., conformity) and the population-level outcomes of these learning biases (e.g., how conformity can generate and maintain between-group cultural variation). A cultural evolutionary approach demands an explanation of multiple social levels (memes, individuals, groups, populations, and societies) and how one level shapes and is shaped by other levels.

Second, there is a fundamental distinction in how fields interpret the term “adaptiveness.” Psychologists often consider a psychological characteristic “adaptive” if it predicts higher self-reported well-being or self-esteem or measures of physical or mental health. Acculturation research is no different, and acculturation strategies are often evaluated in terms of whether they are “adaptive” as in being associated with higher psychological (e.g., well-being, self-esteem, and lack of psychological problems) and socio-cultural adaptation (e.g., ability to productively navigate their cultural environments; Ward & Kennedy, 1994). However, evolutionary approaches to human behavior, including cultural evolution, use the term differently, drawing from evolutionary biology (Laland & Brown, 2011). “Adaptiveness” here refers to whether a behavioral or psychological trait increases an individual’s chances of survival and reproduction (their “fitness”), either currently or in the past. A trait that currently enhances fitness is described as “adaptive.” A trait that originated in the past due to its enhancement of fitness, and may or may not currently be adaptive, is described as an “adaptation.” In a widely adopted framework originated by Mayr (1961), explanations in terms of increased fitness are labeled “ultimate,” while explanations in terms of psychological motivations like self-esteem or well-being are labeled “proximate.”

Third, the social learning strategies that are central to this review are often described as “biases” (e.g., prestige bias) in the cultural evolution literature. “Bias” is not meant here in a normative or moral sense. Rather it means a deviation from randomness, in a statistical sense. For instance, “prestige bias” means a bias to copy prestigious individuals rather than the unbiased copying of other people at random irrespective of their prestige. Hence, there is no suggestion or requirement that prestige bias is morally good or bad, or socially desirable or undesirable.

## Cultural Evolutionary Mechanisms of Acculturation

While the bidimensional acculturation framework assumes that immigrants vary in the extent to which they retain elements of their heritage culture and the extent to which they adopt elements of the dominant majority culture (Berry & Sam, 1997; Ryder et al., 2000), the motivations behind and mechanisms by which this “retention” and “adoption” actually occur are often unspecified. Consequently, a significant task for contemporary acculturation research lies in determining the functional value of diverse cultural styles in different environmental settings. Cultural evolution theory has the potential to fill this gap as it contains numerous specific social learning strategies that describe why, when, and how people learn from one another. One advantage of this perspective is the ability to elucidate cultural transmission in both directions—from the majority to the minority and vice versa given that these social learning strategies should apply broadly, across people and situations, not just to a specific context of immigration. In this section, we zoom in on the first part of our conceptual model (see Figure 1) that deals with how cultural evolutionary modes of cultural transmission influence individual acculturation orientations.

Although the proposed social learning strategies are a common subject of study in cultural evolution, we note that their application and study also permeate other fields. For example, social and personality psychologists have explored the determinants of conformity across various scenarios (Cialdini & Trost, 1998) and the precursors and results of the pursuit of prestige (Maner & Case, 2016). However, this research typically focuses on the proximate psychological mechanisms, processes, and outcomes often within particular social contexts or experimental conditions. It rarely extends to enduring cultural shifts among groups and populations through robust modeling. Nor do these approaches seek to understand the (evolutionarily) adaptive basis of these psychological phenomena to predict when and why such strategies can be expected to be used. By contrast, cultural evolution tackles these phenomena with a distinct focus on deciphering their impact on the transmission and adaptation of cultural attributes, beliefs, and behaviors across, between, and within generations.

### Conformity

Cultural evolution researchers define conformity as being disproportionately more likely to adopt the majority behavior in one’s group or society, compared with copying at random (Boyd & Richerson, 1985). For example, if 60% of people in a society use chopsticks rather than forks, then a conformist entering the society would have a *more than* 60% chance of using chopsticks (see Box 1 for a concrete demonstration of how this process can be modeled). This differs from the classic sense of conformity in social psychology that does not specify disproportionate copying and deals with momentary rather than lasting changes in behavior (Asch, 1951; Cialdini & Trost, 1998). The difference is important because formal modeling shows that only disproportionate and chronic majority copying can generate and maintain strong between-group cultural differences (Boyd & Richerson, 1985). Simply copying the majority (e.g., having an exactly 60% chance of using chopsticks) in a given situation does not lead to any cultural change nor maintain group differences. Experiments have shown that most people tend to conform in this specific sense when faced with an uncertain task (Efferson et al., 2008; Muthukrishna et al., 2016).

**Figure 3.**
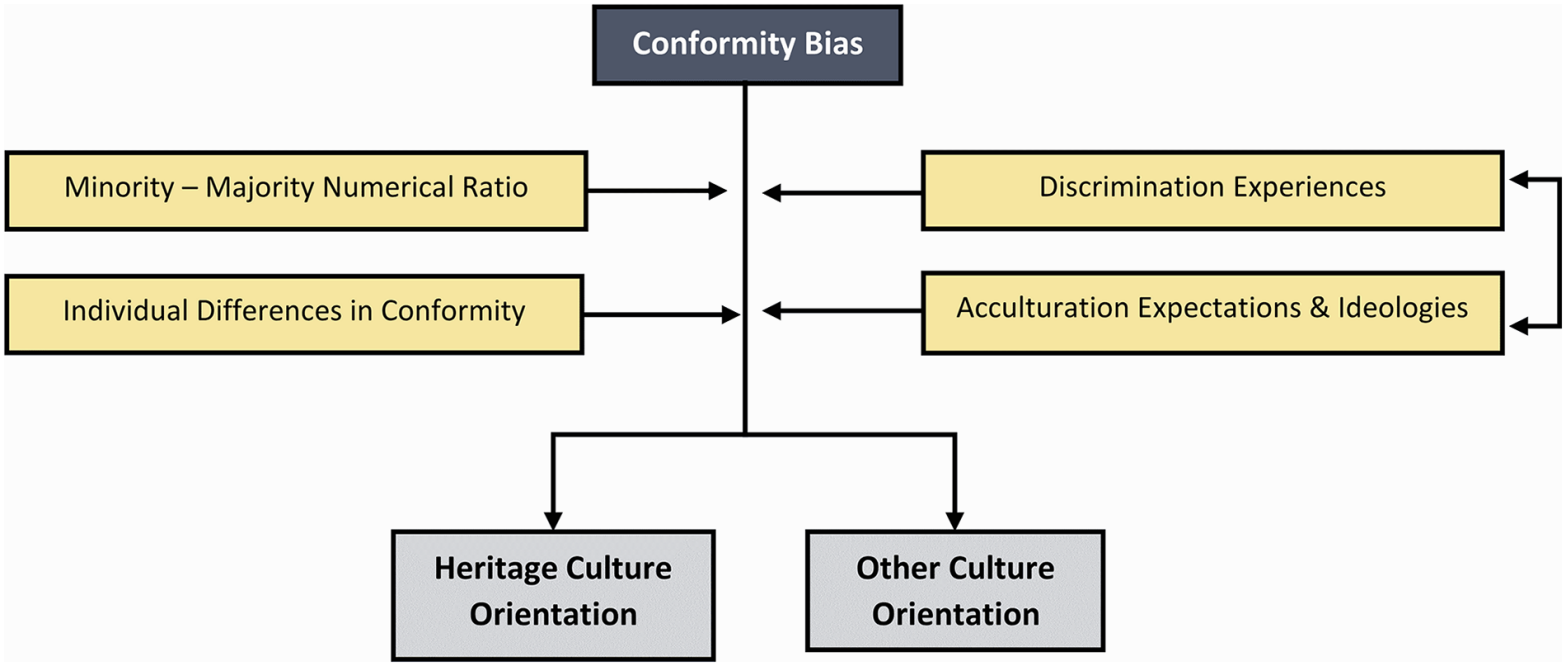
Some of the Potential Factors Moderating the Effect of Conformity Bias.

As hinted with the chopstick example, conformity is a prime candidate for how immigrants and minority-group members may come to adopt cultural traits of the majority given that such traits will typically be more frequent in the destination or mainstream society. Deffner et al. (2020) showed experimentally that participants moving to a new group conformed to the new group’s majority behavior and that this was adaptive given that existing group members had previously learned the optimal behavior in their group and environment. This influence is likely moderated by several factors (see Figure 3). For instance, longitudinally, Nieri (2012) demonstrated how the proportion of ethnic minority individuals within U.S. schools exhibiting characteristics of the dominant culture (i.e., speaking English) positively predicted the adoption of this characteristic over time.

Importantly, however, conformity may also lead to cultural retention among immigrants and minority-group members if the context provides alternative targets for conformity. It is unlikely that people predominantly respond to numerical majority behaviors among the entire population of a country. They also respond to the numerical majority behaviors observed in their local neighborhoods, social networks, and other biased samples from the population. Indeed, in line with theory emphasizing the interaction of group size and conformity (Muthukrishna et al., 2016), immigrants’ values tend to change more toward those of the majority society when living among fewer ethnic peers, but when their ethnic heritage group is larger, this tendency to adopt the majority culture is reduced (Taras et al., 2013). Such a mechanism could explain why conformist tendencies, as commonly assessed through psychometric instruments, frequently predict a higher degree of cultural retention among ethnic minority groups that hold a significant demographic presence in society (Güngör, 2007; Phalet & Schönpflug, 2001).

The acculturation expectations set by the dominant group and prevailing societal ideologies may further modulate the target(s) and strength of conformity bias (see Box 1), establishing varying norms for the adoption of the majority culture and the preservation of heritage culture (Bourhis et al., 1997; Horenczyk et al., 2013). In societies promoting assimilationist principles, where immigrants are strongly encouraged to assimilate, conformity bias may lead to increased adoption of the majority culture compared with societies advocating segregation. However, intense pressures to conform to the majority, coupled with the subsequent marginalization of those who do not conform, may also provoke resistance. This reaction could potentially incite immigrants to distance themselves from the majority culture and intensify their engagement with their heritage culture (Jasinskaja-Lahti et al., 2009; Kunst & Sam, 2013b). The “integration paradox” posits that this propensity is particularly pronounced among highly educated individuals who may be more perceptive of discriminatory practices and ideologies (de Vroome et al., 2014; Verkuyten, 2016). This notion further highlights individual variation in the strength of conformity tied to factors such as socioeconomic status.

Conformity bias can also illuminate the circumstances under which members of a majority group either adopt or reject the culture of immigrants and their degree of commitment to preserving the prevailing majority culture. In societies that are relatively homogeneous with an even geographical distribution of immigrants and minority-group members, conformity bias may typically impel majority-group members to sustain their dominant culture, with low motivation to adopt aspects of immigrant cultures (Haugen & Kunst, 2017). However, as societies become more ethnically diverse due to increased immigration and the emergence of minority-majority dynamics (e.g., minority groups cumulatively forming the majority), conformity bias may increasingly promote the adoption of immigrant cultures among majority-group members. This shift is amplified by immigrants often settling in ethnically diverse areas inhabited by co-ethnic peers. Although the exact thresholds remain to be determined, once these groups reach a certain size, the targets of conformity among majority-group members may change.

Historical analyses support this premise and reflect sociological narratives (P. L. Berger, 2011), indicating that religious conversion rates within cultures are heavily influenced by their initial population sizes—specifically, smaller initial populations expedite the transformation of the minority into majority traits (Watts et al., 2018). This process is accelerated by contact between former majority-group members and converts (Stark & Bainbridge, 1980). Therefore, as the proportion of minority-group members in society rises, conformity bias might propel majority-group members to adopt elements of minority cultures. Conceivably, however, acculturation within the majority group could be contingent on a single minority group achieving numerical majority status, rather than multiple minority groups collectively forming the new majority. Moreover, cultural ideologies also potentially amplify or mitigate the shift of conformity targets toward the minority group (Kunst et al., 2021). While multicultural ideologies may facilitate this shift, ideologies advocating the assimilation of immigrants may incite resistance. This contextual moderation could explain why, in certain settings, majority-group members residing in ethnically diverse neighborhoods are *more* likely to reject minority-group cultures than those living in ethnically homogeneous neighborhoods (Haugen & Kunst, 2017).

### Anti-Conformity

**Figure 4.**
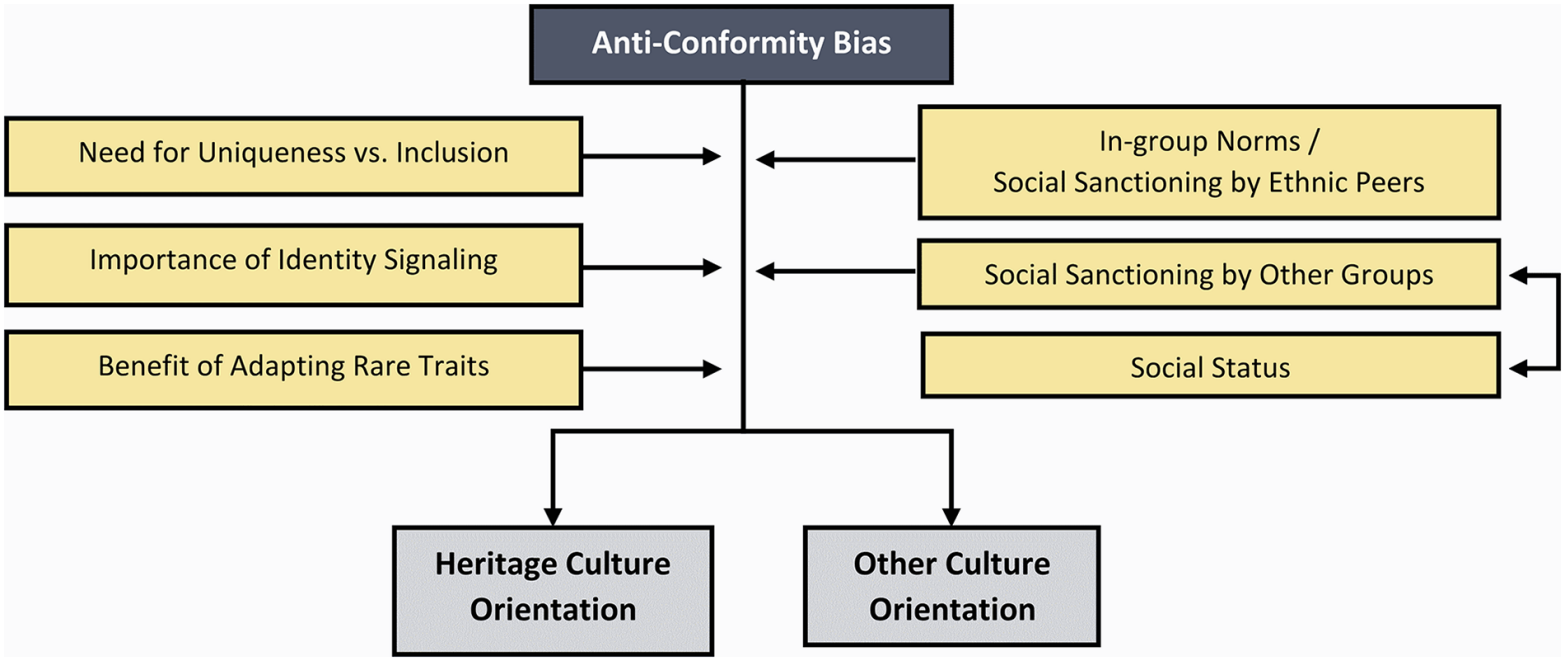
The Potential Factors Moderating the Effect of Anti-Conformity Bias.

The opposite of conformity is anti-conformity: being disproportionately more likely to copy cultural traits that are less common. In the chopstick example earlier, anti-conformists would be *less than* 60% likely to adopt chopsticks (see also Box 1). Anti-conformity may explain minority-group members’ culture retention but could also be a prime candidate for explaining the adoption of immigrant traits by members of the majority (Whalen & Laland, 2015). Historical analyses and simulations confirm the presence of anti-conformity in cultural evolution (Mesoudi & Lycett, 2009; Shennan & Wilkinson, 2001), and social psychological work suggests that the influence of minorities can sometimes outweigh that of majorities (Gardikiotis, 2011; Latané & Wolf, 1981; Moscovici & Nemeth, 1974). Indeed, mathematical modeling directly comparing the influence of conformity to anti-conformity sometimes favors the latter (Eriksson & Coultas, 2009). However, like for conformity, its influence may be moderated by several factors (see Figure 4).

The inclination toward anti-conformity with respect to the majority culture, alongside conformity to one’s ethnic in-group as previously discussed, may explain the heritage cultural retention of some minority groups. Social psychological research indicates humans’ pervasive pursuit of optimal social distinctiveness, that is, balancing the need for both uniqueness and social inclusion (Brewer & Roccas, 2001; Leonardelli et al., 2010). These twin motives may prompt minority group members to exhibit at least some degree of anti-conformity, enabling them to retain elements of their heritage culture. The existence of anti-conformity norms could further bolster this tendency by instigating social penalties for non-compliance. Indeed, empirical studies investigating the phenomenon of “intragroup marginalization” provide compelling evidence of this (Castillo, 2009; Castillo et al., 2007, 2008), revealing that immigrants and minority-group members perceived as adopting the majority culture risk ostracism from their ethnic peers. Simultaneously, anti-conformity also may incur sanctions from the majority group, particularly when assimilation is anticipated (Kosic et al., 2005; Piontkowski et al., 2002). While higher status immigrants, like sojourners, might be less affected by such sanctions, members of lower status and vulnerable groups, including refugees, may be particularly susceptible to this punitive action as they are often expected to assimilate (Montreuil & Bourhis, 2001, 2004; Tahir et al., 2023). This disparity potentially restricts the degree to which certain groups can realistically afford anti-conformity.

The anti-conformity motives prevalent among minority groups align with research exploring the rejection of cultural traits and practices through “identity signaling” (J. Berger & Heath, 2008). Cultural traits serve as pivotal markers of group identities, acting as adaptive heuristics that guide individuals toward potentially productive and trustworthy interactions (Brewer, 1999). By preserving minority cultural traits rather than adopting those of the majority, minority-group members effectively signal their identity to others, which can ensure important outcomes such as social support.

The demographic proportions of immigrants, minority-group members, and majority-group members are in flux and may recalibrate majority groups’ conformity targets toward immigrants as previously noted. Nevertheless, anti-conformity could explain majority-group members’ uptake of immigrant cultures, particularly when their majority group represents the unequivocal numerical majority across most contexts and settings. The principle of optimal distinctiveness may again elucidate this anti-conformity inclination among majority-group members. Research indicates that a considerable portion of majority-group members perceive themselves as “excluded from multiculturalism” (Plaut et al., 2011; Rios & Mackey, 2022). Thus, the incorporation of cultural elements from other groups and giving up parts of the majority culture may satiate this felt need for uniqueness (also see Inesi & Rios, 2023), fostering a sense of inclusion and belonging. Alternatively, it may be that anti-conformity is a strategy followed by low-status members of the majority as a means of acquiring rare and possibly beneficial traits not known by most others, as a pursuit of gaining social standing (Reader & Laland, 2001).

### Payoff Bias

Payoff bias describes the tendency to copy cultural traits associated with high payoffs, in terms of fitness or proxies of fitness such as wealth or calorie returns. Experiments show that most people preferentially copy behaviors or choices associated with higher payoffs than their current behavioral defaults (McElreath et al., 2008; Mesoudi, 2011b; Morgan et al., 2012). This tendency is also consistent with behavioral economics, where people are broadly assumed to maximize their utility, albeit within limits set by cognition (Camerer, 1999), as well as a dynamic constructivist approach to culture and cognition (Hong et al., 2000, 2001), positing that cultural knowledge operates as a toolkit.

**Figure 5.**
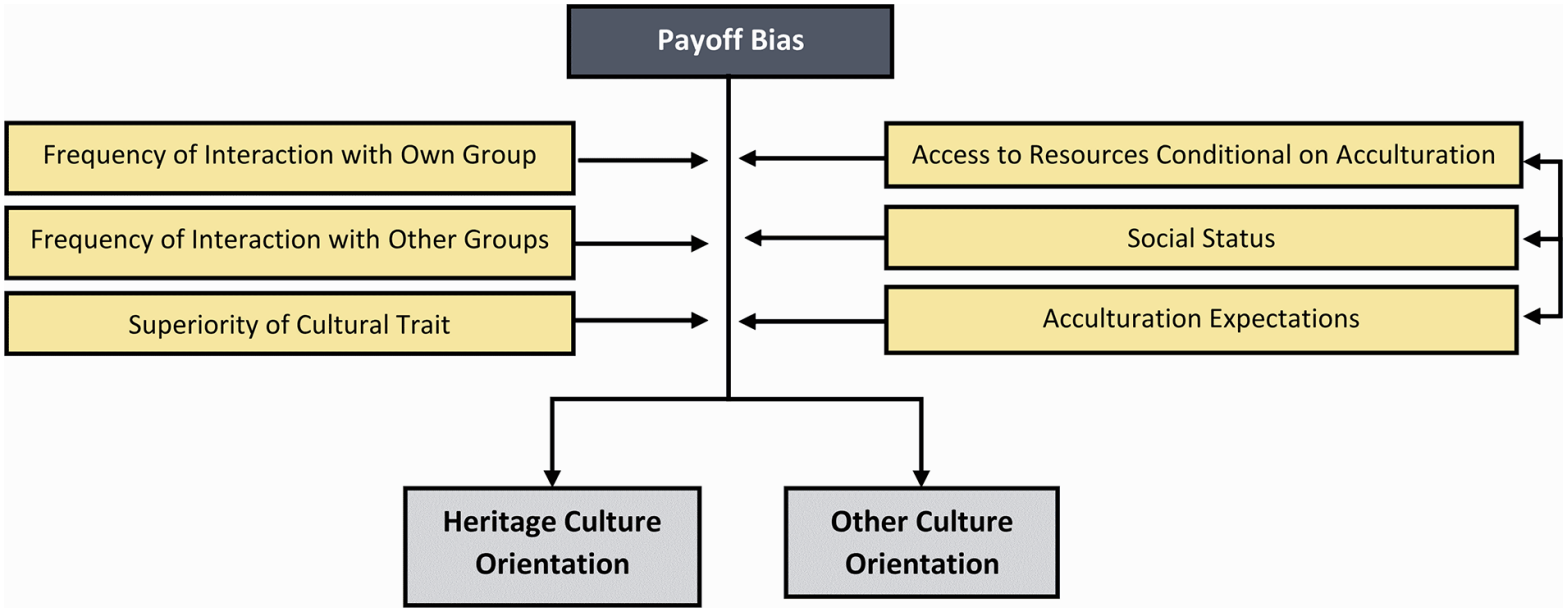
The Potential Factors Moderating the Effect of Payoff Bias.

In terms of minority group acculturation, coordination payoffs and conditional access to resources may favor the adoption of traits from the majority group via payoff bias (see Figure 5). If interactions between individuals yield higher payoffs when both individuals possess the same cultural values (e.g., both are relatively individualistic), then immigrants may be driven to adopt traits from the majority group to improve their payoffs from such interactions. Generally, this payoff perspective is consistent with interactive models of acculturation (Bourhis et al., 1997; Horenczyk et al., 2013) that predict more productive intercultural relations and interactions when there is a consensus between societal ideologies, majority-group members’ expectations toward the minority group, and the actual acculturation preference of the minority group. Conversely, a mismatch between the majority and minority cultural traits (e.g., an individualistic individual interacting with a collectivistic individual) may lead to coordination loss, friction, and conflict (Piontkowski et al., 2002).

Empirical research on person-organization fit (Cable & Judge, 1996; Charles et al., 1991) supports the role of payoff bias in the acculturation of minority groups. For example, the alignment of an individual’s social values with those prevalent in mainstream society positively predicts desirable employment outcomes such as job tenure (Parkes et al., 2001). Similarly, among expatriate workers, a perceived congruence between the team’s values and one’s own has been associated with reduced intentions to leave the job and superior work adjustment (Choi et al., 2017).

Ethnocentric tendencies exhibited by key personnel, such as managers and human resources professionals, may amplify the immediate benefits of embracing the majority culture. Experimental evidence indicates that employers from the majority group preferentially hire members from minority groups who seem to have adopted the majority culture. For instance, Gürlek (2020) reported a 60% employment rate for assimilated applicants, a stark contrast to 23% for separated and 42% for integrated applicants. Correspondingly, hiring managers have been found to appraise person-organization fit and employability most favorably for applicants who have incorporated the majority culture into their private lives (Bye et al., 2014; Horverak et al., 2013). Thus, from a payoff perspective, it may in many contexts be adaptive for minority-group members to adopt the majority culture. Importantly, the social status of immigrants and minority-group members can significantly moderate the payoffs of adopting the majority culture, especially in environments where assimilation is generally expected (J. Berger, 2008). For higher-status groups like sojourners and skilled migrants, who have abundant job opportunities and/or are perceived as culturally akin to the majority, the payoffs of adopting the majority culture might be less pivotal. Conversely, for low-status and vulnerable groups, the impetus to assimilate into the majority culture might be intensified due to the substantial payoff it yields.

However, payoff bias may also explain why many immigrants and members of minority groups choose to preserve aspects of their heritage culture. A robust affiliation with positively-valued groups is postulated to augment well-being via enhanced collective self-esteem (Tajfel, 1978, 1982). Accordingly, a strong social identity is linked to increased satisfaction of needs (Greenaway et al., 2016), including social support (Häusser et al., 2023; Junker et al., 2019), is positively correlated with (but possibly not predictive of) self-esteem (Phinney & Chavira, 1992; but see Umaña-Taylor et al., 2008) and may potentially provide resilience against stressors such as ethnic discrimination (Branscombe et al., 1999; Cronin et al., 2012). As such, maintaining one’s ethnic heritage identity may have several payoffs for minority-group members. Nevertheless, social identity constitutes only one domain of acculturation, and recent longitudinal meta-analytical research and studies within the acculturation literature have prompted skepticism about the positive repercussions of preserving one’s heritage culture for the typical markers of well-being and social support (Bierwiaczonek & Kunst, 2021; Zagefka et al., 2023). And from a (cultural) evolutionary perspective, well-being and self-esteem would be classed as proximate rather than ultimate explanations (see above). What then could be the alternative immediate payoffs for upholding one’s cultural heritage?

Particularly in contexts that involve frequent interactions with co-ethnics and societies that appreciate diversity, maintaining one’s heritage culture could potentially yield resource benefits (Jones Christensen & Newman, 2022). Business owners who preserve their heritage culture might effectively engage with customers of the same ethnic group, productively function as intercultural mediators, and forge both national and international business relationships, especially with individuals sharing the same cultural heritage. In addition, a network of co-ethnics could offer job seekers access to employment opportunities, especially for those with low status and a lack of opportunities elsewhere (e.g., refugees and undocumented immigrants). Businesses providing culturally authentic services may increase their profits by appealing to majority and minority customers alike.

Importantly, payoff bias may also explain why members of the majority group might incorporate cultural aspects from immigrant and minority groups. If a trait brought by an immigrant into a society is more effective than existing alternatives, then it may spread through the entire society. Although rarely studied in the field of acculturation (but common in cultural evolution), technology provides a good example of this, with inventions from the telephone to the internet resulting from knowledge brought by immigrants. The diffusion of innovations literature (Rogers, 1995) contains examples of this process, with caveats such as that the innovation spreads best if it resembles in some way the existing alternative, if it can be easily observed or tested, and if recombined or blended with existing traits to make it more palatable to the majority.

Revisiting the religious conversion example from the “Conformity” section, embracing aspects of immigrants’ and minority-group members’ culture such as their religious beliefs could yield additional immediate payoffs for members of the majority group. A host of meta-analyses have highlighted the positive correlation of religiosity with both psychological and physical well-being (Hackney & Sanders, 2003; Jim et al., 2015), although longitudinal effects are weaker (Garssen et al., 2021). In increasingly individualistic, secular, and atheistic societies, religions such as Islam may cater to a rising demand for a sense of meaning and community (Snook et al., 2021; Zebiri, 2014). Furthermore, from a business perspective, aligning with the cultural practices of environments dominated by minority-group members provides immediate payoffs for majority-group members. For instance, in neighborhoods with significant Muslim or Jewish populations, offering halal or kosher-compliant products and services can considerably expand the customer base.

Finally, adopting aspects of immigrant and minority-group cultures can also confer everyday benefits on majority-group members, particularly those living in culturally diverse neighborhoods (Kunst et al., 2021). Such adoption promotes a sense of “cultural fluency” (Oyserman, 2011), recently introduced as an alternate marker of adjustment within the acculturation literature (Doucerain, 2019). Cultural fluency refers to the alignment between someone’s cultural knowledge and their environment, resulting in automatic behavior and expectations that typically fit with daily situations, thereby minimizing friction. As per the fitness-maximization perspective that informs evolutionary understandings of payoff bias, cultural fluency may be adaptive as it allows for optimized expenditure of energy and resources while traversing through one’s cultural milieu.

### Prestige Bias (and Other Model-Based Biases)

While payoff bias concerns the payoffs derived from the cultural trait being copied, model-based biases describe tendencies to copy traits based on the characteristics of the bearers of those traits, independent of the trait itself. We focus particularly on prestige bias, which describes a tendency to copy individuals who have high social status (Brand et al., 2020; Henrich & Gil-White, 2001; Jiménez & Mesoudi, 2019), while other model-based biases include copying people who are older or of the same gender. Model-based biases are typically viewed as indirect, less costly means of acquiring high-payoff cultural traits, whereas the more direct payoff bias (see previous section) is costly or difficult. For example, generally copying a prestigious soccer player like Kylian Mbappé or a successful businessman like Elon Musk^1^ is often easier than figuring out exactly what specific physical training regime or entrepreneurial strategies led to those individuals’ social success in the first place. While easier, this runs the risk of copying irrelevant or even maladaptive traits that did not actually lead to their success, such as their hairstyle or clothing, or attempting to copy individuals whose success is uncopyable, for example, having rich or socially connected parents, illustrating the trade-offs inherent in acquiring adaptive cultural behaviors.

**Figure 6.**
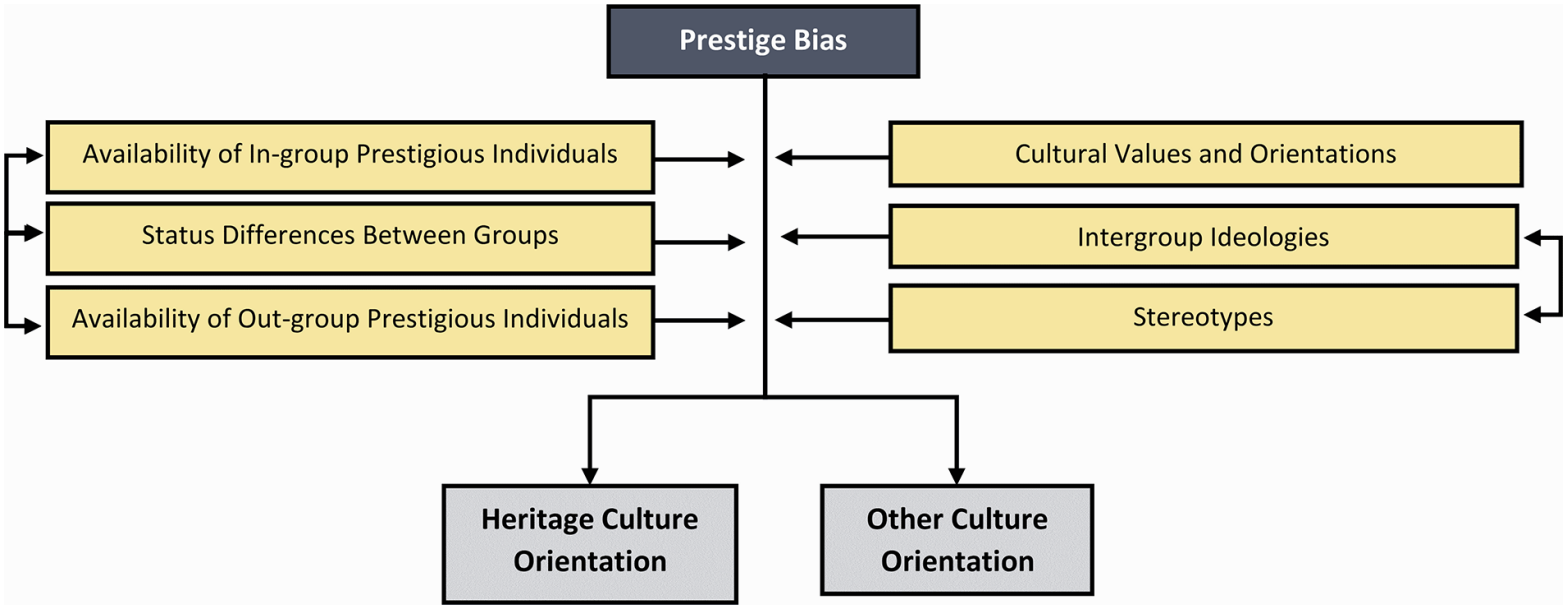
The Potential Factors Moderating the Effect of Prestige Bias.

Prestige bias provides another mechanism by which immigrants and minority groups can adopt mainstream culture if they preferentially copy prominent teachers, celebrities, sportspeople, and other prestigious figures from the majority group (see Figure 6). Importantly, however, prestige bias is critically influenced by historical and socio-political structures; there is no “objective” standard of prestige, it is always culturally and historically contingent. Given the prevailing inequality in most culturally diverse societies, societal status is often disproportionately conferred to the majority group (Sidanius & Pratto, 1999). This imbalance suggests that, et ceteris paribus, more individuals within this group would be deemed prestigious and serve as influential sources of learning. Prestige bias in such unequal settings emerges as a social mobility strategy, particularly for lower status groups (Ellemers, 1993; Ellemers et al., 1993; Samnani et al., 2013). This perspective also aligns with empirical findings showing that lower-status groups tend to demonstrate less in-group favoritism and occasionally exhibit a preference for high-status out-groups (Sidanius & Pratto, 1999).

Individual variability in prestige may also shape immigrants’ and minority-group members’ maintenance of their heritage culture. If members of an ethnic (minority) in-group occupy particularly esteemed societal roles—for example, as politicians, sportspeople, or entrepreneurs—this could foster ethnic pride and encourage engagement with one’s cultural heritage. Notably, the influence of role models on minority-group members’ acculturation has been scarcely examined and represents a promising line of research stemming from a cultural evolution perspective. Nevertheless, existing evidence suggests that positive role models within one’s ethnic group—such as extended family or community members—may be associated with enhanced preservation of heritage culture, a more stable ethnic identity, and occasionally less adoption of the majority culture (Knight et al., 2010; Phinney & Ong, 2007; Yancey, 1998). Importantly, however, negative role models may also sway acculturation orientations and strategies. This may be particularly salient for individuals from collectivistic backgrounds for whom avoiding the path of negative role models may provide stronger motivation than following positive role models (Lockwood et al., 2005). Thus, ethnic in-group members of low social standing might inspire either increased or decreased maintenance of heritage culture among immigrants and minority-group members, contingent upon whether this culture is perceived to have contributed to their circumstances.

Although not investigated sufficiently to date, prestige bias may have similar impacts on the acculturation of majority-group members. As immigrants ascend the social hierarchy, achieving esteemed positions within society, majority group members may implicitly learn from these successful individuals. Circumstantial evidence at the group level supports this. In a set of studies by Kunst, Bierwiaczonek, et al. (2023), the more competent, warm, and moral immigrants were perceived to be, the greater the motivation was for majority-group members to adopt their culture. Although such stereotypes are only a proxy of prestige, these mechanisms likely operate in the context of inter-individual cultural transmission as well. Further bolstering this idea is research concerning acculturation expectations, which clearly indicates that majority-group members exhibit a higher level of support for immigrant groups maintaining their culture the higher societal status the groups hold (Kunst & Sam, 2014; Montreuil & Bourhis, 2001, 2004).

Conceivably, the prestige of in-group members might shape the acculturation of majority group members, similar to its effect on minority groups. To illustrate, if a popular political figure within the ethnic majority adopts elements of immigrant culture, this could prompt followers to emulate this acculturation orientation. For instance, Jacinda Ardern’s adoption of Māori customs, such as wearing a Korowai when meeting the Queen (Illmer, 2018), may inspire other White New Zealanders to also engage in this culture. Conversely, if a prestigious figure espouses nationalist ideologies, it might lead followers to resist or reject the culture of immigrants and minority-group members (Crandall et al., 2018; Kunst et al., 2019). Importantly, the influence of prestige extends beyond the political sphere, permeating domains such as sports and entertainment. For example, the conversion of celebrated boxer Muhammad Ali (formerly Cassius Clay) or musician Yusuf Islam (formerly Cat Stevens) to Islam may have sparked curiosity and interest among their fanbases, potentially prompting some to explore, and perhaps even adopt, the faith themselves.

### Vertical Cultural Transmission

**Figure 7.**
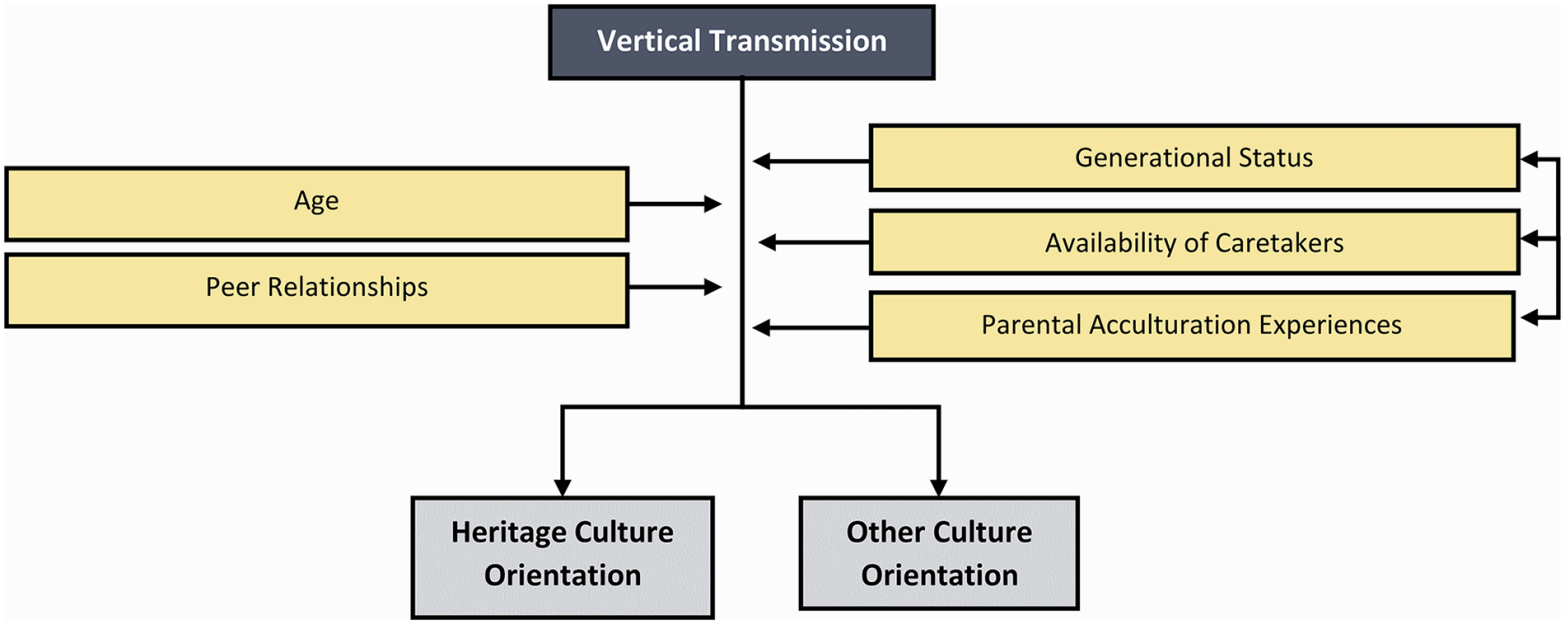
The Potential Factors Moderating the Effect of Vertical Transmission.

A special case of cultural transmission is learning from one’s parents, labeled “vertical” in the cultural evolution literature because it involves the flow of information down the generations, in contrast to “horizontal” which involves social learning within generations (Cavalli-Sforza & Feldman, 1981). Conformity or prestige biases are not viable when one’s only potential sources of information are one or two parents. This is why research suggests that while parents are initial sources of social information, by adolescence people primarily learn from other members of society (Aunger, 2000; Harris, 1995; Henrich & Broesch, 2011; Verkuyten et al., 2012). Nevertheless, vertical cultural transmission is of special interest for acculturation research because an immigrant’s or minority-group members’ parent(s) will often possess cultural traits different from the majority-group culture within which they grow up (see Figure 7).

Several studies highlight the influence of vertical cultural transmission from immigrant parents to their children. Generally, family socialization primarily predicts more cultural maintenance (Huijnk et al., 2012), but nuances exist. In samples of immigrant parent–child dyads, parental collectivism values were passed down but not individualism (Phalet & Schönpflug, 2001). Similarly, variables associated with vertical cultural transmission such as family contact are often stronger predictors of collectivism, whereas measures related to horizontal cultural transmission such as mainstream media use and years of mainstream education are stronger predictors of individualism (Mesoudi et al., 2016).

More generally, the children of immigrants are typically found to be intermediate between the heritage cultural values of their parents and the local cultural values of majority-group members (Bell, 2013; Dinesen & Hooghe, 2010; Mesoudi et al., 2016; Uskul et al., 2011), indicating a mix of vertical and horizontal transmission. For instance, although the mate preferences and acculturation orientations of immigrants are predicted by those of their immigrant parents, differences are observed, especially in the public domain (Costigan & Dokis, 2006; Hynie et al., 2006). Furthermore, generalized trust of immigrants is the result of parental socialization as well as perceptions of the majority society (Dinesen, 2012). Future work might integrate theoretical models predicting when and for what kind of traits vertical cultural transmission should play a role (McElreath & Strimling, 2008) and model-selection-driven statistical techniques for examining specific markers of vertical transmission (e.g., time spent with parents, parents’ individual differences such as homesickness or social status; Spiegler et al., 2016, 2019) versus horizontal transmission (e.g., exposure to mainstream mass media and majority contact; Mesoudi et al., 2016).

Vertical cultural transmission processes intersect with the individual characteristics of minority-group members and immigrants. Adult immigrants typically encounter the majority of vertical transmission prior to their migration experience. Conversely, the cultural orientations that immigrant or subsequent-generation parents convey to their children, born in the host country, may be significantly shaped by both their personal and their group’s experiences in the society of residence. Unaccompanied child migrants may experience a disruption in vertical cultural transmission upon migration. Some may be assigned new caretakers, and the nature of these relationships can lead to substantial changes in the culture that is vertically transmitted and also amplify the role of horizontal transmission from peers (Omland & Andenas, 2020; Omland et al., 2021).

Vertical cultural transmission is not exclusive to minority groups (see Kwak, 2003). Indeed, cultural traits and acculturation orientations among majority-group parents likely also propagate to their offspring. Although acculturation research is needed here, studies in the related field of intergroup relations demonstrate that the social dispositions of majority-group members are partly parentally influenced. Seminal contributions (Allport, 1954; Bandura & Walters, 1977; Frenkel-Brunswik, 1948) and comprehensive longitudinal meta-analyses (e.g., Crocetti et al., 2021) indicate that individual intergroup orientation can be linked back to parental attitudes. Consequently, majority-group members’ acculturation strategy (e.g., integration or separation) may be transmitted to their children.

## Acculturation Strategies as Cultural Evolutionary Equilibria

As outlined above, at least five cultural evolutionary mechanisms can potentially explain the extent of cultural maintenance and adoption among immigrants and minority-group members as well as majority-group members. Here, we outline how these mechanisms collectively can manifest in the different acculturation strategies (integration, assimilation, separation, and marginalization) reviewed earlier. Acculturation psychologists assume that societies can be characterized by individuals exhibiting acculturation strategies at certain frequencies. Among minority groups, evidence generally indicates that the integration strategy is most frequent, followed by either assimilation or separation, then marginalization (Berry et al., 2006b; Bierwiaczonek & Kunst, 2021). Among majority-group members, integration is also observed to be one of the most frequent strategies, alongside separation (Kunst et al., 2021). We suggest that the given distributions of acculturation strategies can be viewed loosely as mixed equilibria at the level of the society—equilibria that result from the cultural evolutionary mechanisms discussed above operating within the context of certain environmental, group, and individual determinants (see mid-section of Figure 1). While our use of the term “equilibrium” here is meant in a relatively informal sense, future modeling work might formalize it to determine the actual stability of population-level acculturation strategy combinations under different conditions, whether they form stable or unstable equilibria, and so on.

This notion highlights that while individuals can pursue acculturation strategies, as typically studied in acculturation psychology, we also need to understand the population-level combination of strategies. This is especially the case if strategies interact in a frequency-dependent manner. For example, immigrants might be more likely to pursue separation if there are many other members of the immigrant community who are already pursuing this strategy. Simultaneously, this may cause majority-group members to be less likely to pursue integration. These population-level considerations naturally stem from a cultural evolution framework, which has long explored how individual and population levels interact.

We stress that different acculturation equilibria are unlikely to be the outcome of just one cultural evolution mechanism. Rather, they almost certainly result from a combination of them favoring different strategies for different groups, depending on contextual and individual factors. Thus, to understand the forces that favor certain population-level cultural equilibria, it is essential to understand what cultural evolutionary mechanisms favor each strategy. In the following subsections, we first outline exactly how the cultural evolutionary mechanisms described earlier might favor or disfavor the four acculturation strategies, contributing to the cultural evolutionary equilibria seen in different societies. The subsequent section then explores the implications of these equilibria at the population level.

Acculturation strategies here are assumed to reflect the behaviors that individuals exhibit, rather than their preferences. Our interest lies in behavior because it serves as the most direct catalyst for cultural change from the standpoint of cultural evolution. However, this distinction is far from trivial. Individuals belonging to stigmatized minority groups who might prefer a certain strategy, such as integration, might be precluded from realizing this preference due to discrimination, exclusion, structural barriers, and their generally lower power in society (Berry, 2003, 2005; Verkuyten & Martinovic, 2012). Furthermore, in the following sections, it is crucial to remember that the same acculturation strategy may be observed among minority and majority-group members, albeit with a mirrored interpretation. For instance, the strategy of assimilation among immigrants indicates a propensity to relinquish their heritage culture and adopt the culture of the dominant majority group. Conversely, among majority-group members, assimilation signifies a tendency to forgo the dominant majority-group culture in favor of the cultures of immigrants and minority groups. While both groups may exhibit the same acculturation strategies driven by similar cultural transmission processes, they may often be influenced asymmetrically by contextual cues.

### Integration

**Figure 8.**
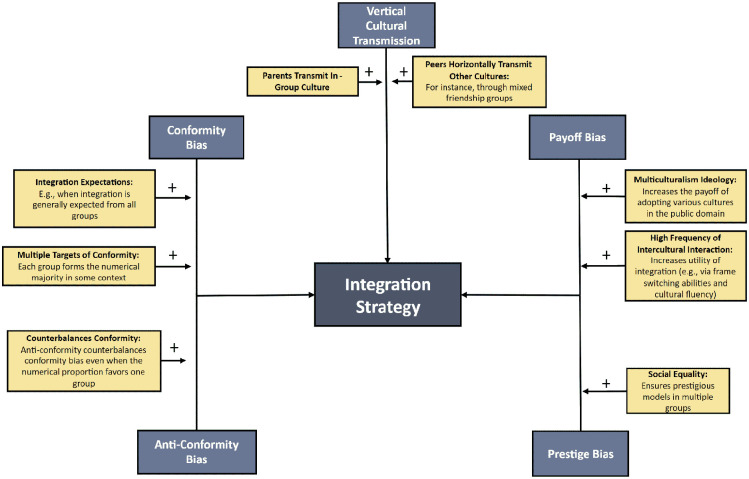
Possible Scenarios in Which Cultural Evolutionary Processes Manifest in the Integration Strategy.

Integration may largely stem from cultural evolutionary processes that cause individuals to simultaneously participate in different cultural spheres (see Figure 8). In terms of conformity bias, integration may occur when both the in-group and out-group(s) represent a certain size, thereby forming multiple targets eliciting conformity (Muthukrishna et al., 2016). Importantly, these targets may vary depending on the level of analysis. For instance, within a particular neighborhood, a national minority might be the numerical majority, thereby encouraging conformity to the minority culture among both majority- and minority-group members residing in the area. However, at the national level where the majority group may be most salient, conformity bias supports the persistence of the majority culture (for majority-group members) or its adoption (for immigrants and minority-group members).

Interestingly, even in scenarios where the national majority group is the numerically largest group across various societal levels, an anti-conformist bias could favor integration strategies. While people on average exhibit a tendency toward conformity, there is significant individual variation in this propensity (Cialdini & Trost, 1998; Efferson et al., 2008; Hodges, 2017) and likely also in anti-conformity. In addition, group factors such as acculturation expectations for the minority and majority may modulate these tendencies (Bourhis et al., 1997; Castillo et al., 2008; Kunst, Ozer, et al., 2023). Environments that expect integration from all groups may favor simultaneous engagement in different cultural spheres. Therefore, integration is likely due to the combination of individual and contextual conformity-modulating factors.

Payoff bias may favor integration, particularly in societies that view diversity as an asset, as embodied by the multiculturalism ideology (Berry et al., 1977; Stogianni et al., 2023) or polyculturalism (Rosenthal & Levy, 2012). In such environments, cultural versatility may yield better outcomes in domains such as labor markets, politics, entertainment, and entrepreneurship. For instance, job seekers may be more successful when they showcase competence in various cultures. Indeed, the ability to switch cultural frames improves the accessibility of contextually relevant information (Hong et al., 2000; Schwartz et al., 2017; Shih et al., 2010). Moreover, in culturally diverse neighborhoods, integration may also convey fitness benefits by facilitating coordination (Doucerain, 2019).

In situations where both minority and majority groups produce high-status individuals, prestige bias is likely to support social learning outcomes that align with integration. Contextual factors such as racial and ethnic inequality may play a decisive role in determining whether prestige bias favors integration. For example, in highly unequal societies, it can be challenging for immigrants and minority-group members to ascend social ranks to a degree that renders them prestigious enough to stimulate the preservation or adoption of their culture. Note, however, that prestige can take many forms, and low-status groups might engage in social creativity strategies that redefine the criteria for evaluating their social group (Tajfel & Turner, 1979). For instance, in circumstances marked by inequality and oppression, prominent figures from low-status groups, such as civil rights activists, may be perceived as prestigious and motivate engagement in the culture of low-status marginalized minority groups.

Finally, integration may reflect the vertical transmission of one’s ethnic in-group culture and parental preferences for engagement in it combined with the horizontal transmission of cultural elements from other groups (e.g., through socialization; Karataş et al., 2023). Alternatively, simultaneous horizontal and vertical transmission of multiple cultural spheres (as when parents and friends engage in several cultures at the same time and themselves follow the integration strategy) could also foster integration. However, it is vital to distinguish between the private and public spheres of acculturation. Some immigrants may practice integration in the public sphere but separation in the private sphere (Arends-Tóth & van de Vijver, 2007; Rojas et al., 2014). Consequently, horizontal and vertical cultural transmission may exert divergent influences on these two spheres.

### Separation

**Figure 9.**
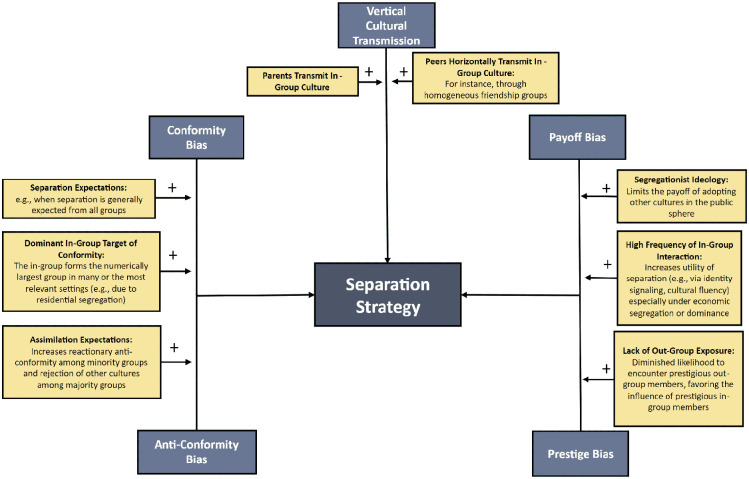
Possible Scenarios in Which Cultural Evolutionary Processes Manifest in the Separation Strategy.

Separation may be interpreted as an outcome of evolutionary mechanisms that prioritize the maintenance of one’s ethnic in-group culture over those of other groups (see Figure 9). Conformity bias may favor separation when a person’s ethnic in-group is socially most salient and forms the numerical majority target of learning. Such circumstances may arise due to residential segregation, resulting in minimal and often negative interactions between minority and majority groups (Kunst et al., 2021). Cultural ideologies, including assimilation and experiences of ethnic discrimination, may lead minority groups to disconnect from mainstream society via anti-conformity and align more closely with their ethnic in-group (Christ et al., 2013; Jasinskaja-Lahti et al., 2009; Kunst & Sam, 2013b; Verkuyten & Yildiz, 2007). Such ideologies might also persuade the majority group to disregard immigrant cultures while perceiving their own as the norm (Devos & Banaji, 2005; Kunst et al., 2018). These mindsets, ideologies, and norms could encourage both groups to favor in-group conformity, sustaining their respective cultures and limiting cultural exchange.

Regarding payoff bias, when the advantages of preserving one’s ethnic in-group culture outweigh the benefits of adopting other cultures, separation becomes more likely. For example, if immersion in one’s culture yields immediate benefits such as expanded co-ethnic networks and career opportunities, and employers prefer employees from their ethnic in-group, then signaling one’s cultural prototypicality by maintaining one’s culture and disregarding others may optimize rewards (J. Berger, 2008; J. Berger & Heath, 2008). These tendencies might be modulated by factors such as inequality and societal norms. In segregated societies with partly parallel economies, adhering to one’s culture might offer higher rewards than in societies where resources are controlled predominantly by another group. In typical scenarios where the majority group maintains most control over resources, norms encouraging assimilation from minority groups may enhance separation among majority-group members, as this becomes the acculturation strategy with the highest rewards. Meanwhile, it may weaken separation tendencies among minority groups who appear compelled to adopt the majority culture for economic survival. Socially, if an individual mainly interacts with in-group members, the immediate benefits of adopting other cultures would be marginal. Instead, cultural fluency (Oyserman, 2011) is predominantly cultivated by engaging in one’s in-group culture in such environments. Intergroup norms and ideologies (e.g., segregationism) that deter cultural exchange through social sanctioning might further enhance the benefits of adhering to one’s in-group culture (Castillo et al., 2008; Kunst et al., 2022).

In relation to prestige bias, contexts that lack exposure to other groups might lead individuals to identify with role models from their own ethnic group, reinforcing the preservation of in-group culture and impeding the adoption of other cultures. Social inequality may further amplify this propensity among majority-group members, while potentially diminishing it among minority-group members. If ethnically stratified social inequality leads to more majority-group individuals with elevated prestige, cultural preservation among this group could be reinforced. However, exposure to these prestigious individuals could alleviate separation tendencies among minority-group members, prompting them to emulate successful majority-group individuals.

Finally, the adoption of separation could be the result of particularly strong vertical cultural transmission and low horizontal transmission, provided that parents primarily transmit and maintain their ingroup culture. Separation may also result from the simultaneous biased horizontal (e.g., through friendship groups that primarily consist of ethnic in-group members) and vertical cultural transmission of the ethnic in-group’s culture, with less emphasis on the culture of other groups (Güngör et al., 2011; Kunst et al., 2021; Phalet & Schönpflug, 2001; Szabó et al., 2020).

### Assimilation

**Figure 10.**
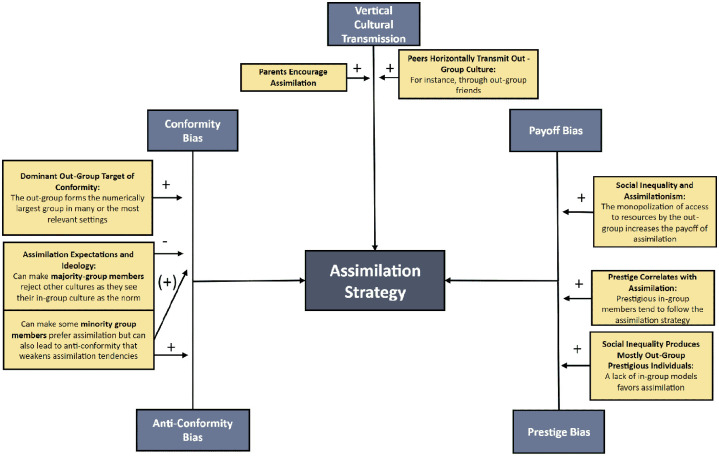
Possible Scenarios in Which Cultural Evolutionary Processes Manifest in the Assimilation Strategy.

Assimilation (see Figure 10) is frequently observed among minority-group members yet is seldom seen among majority-group members (Berry et al., 2006b; Kunst et al., 2021; but see Dandy et al., 2023). This discrepancy aligns logically with a cultural evolution viewpoint. In the context of conformity bias, despite increasing cultural diversification, the historical majority group remains the numerically largest and most influential group in most societies. Consequently, despite the geographical variation in group sizes covered earlier, the overarching effect of conformity might compel at least some minority-group members to align more with the majority culture than with their minority culture (Mesoudi, 2018). Such individuals might simultaneously exhibit low anti-conformity, which may differentiate them from those who react to assimilative pressures by separating.

Although assimilation has been rarely observed among majority groups, it would be intriguing to investigate if majority-group members will increasingly follow assimilation in the future. Indicative of this, a study in Australia reported a cluster of majority-group members inclined toward the culture of immigrants and minority-group members, distancing themselves from their heritage group (Dandy et al., 2023). The ongoing demographic shifts in many immigrant-receiving countries may make such assimilated clusters of majority-group members more likely in the future due to conformity bias shifting its target.

Regarding payoff bias, societal inequality leading to asymmetric resource distribution and assimilative norms could favor assimilation among some minority-group members. For example, when access to resources is monopolized by the out-group and where cultural assimilation is the norm and thereby enhances success (e.g., in the labor market, housing market, friend acquisition, mating), some minority-group members may be driven to relinquish their heritage culture in favor of the majority culture. However, for majority-group members, the tangible benefits of assimilation to minority-group cultures could be limited or even detrimental in such contexts due to intragroup marginalization (Kunst et al., 2022).

Regarding prestige bias, societal inequality that allows more majority- than minority-group members to reach high-status positions in society may encourage assimilation among minority groups. In addition, the cultural orientations of prestigious in-group members may be influential. If successful minority role models publicly portray themselves as assimilated individuals, they could potentially inspire other minority-group members to imitate this acculturation strategy, especially if they perceive it as contributing to the role model’s success. Despite majority groups typically occupying the most prestigious positions, many minority-group members overcome structural adversity to attain such positions. Yet given the skewed numerical proportion relative to prestigious majority-group members, they may be more likely to inspire integration rather than assimilation among majority-group members.

Finally, relatively stronger horizontal than vertical transmission may favor assimilation. Horizontal transmission will often result in learning from members of the out-group rather than one’s parents, who most often will be members of the ingroup. However, this may not always be the case. Some first-generation immigrant parents, particularly those from low-status groups, may foster cultural assimilation in their children in the hope of bolstering their social and cultural capital (Kempny-Mazur, 2017), although these children sometimes exhibit less assimilation than their parents (Birman & Trickett, 2001; Verkuyten, 2016). For effective vertical transmission of assimilation, it is likely that horizontal transmission must reinforce vertical transmission. Similar patterns might underpin assimilation among majority-group members as well. For example, socialization in neighborhoods where national ethnic majority-group members are not the numerical majority, coupled with parental orientations toward cultures of other groups and low levels of ethnocentrism, could foster assimilation.

### Marginalization

**Figure 11.**
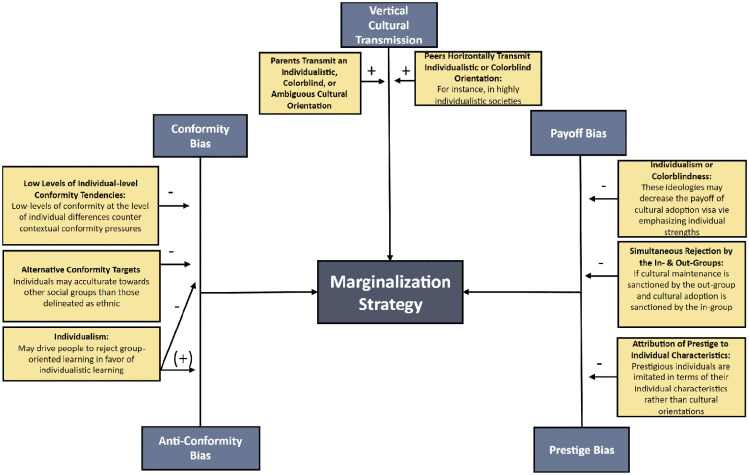
Possible Scenarios in Which Cultural Evolutionary Processes Manifest in the Marginalization Strategy.

Marginalization (see Figure 11) is the least prevalent among minority-group members (Berry et al., 2006b). It is also rare among majority-group members, although certain individuals who do not clearly fit into one of the four designated acculturation strategies might exhibit traits akin to marginalization (usually referred to as the “diffuse” acculturation cluster; Kunst et al., 2021). Individuals who adhere to marginalization may demonstrate particularly low levels of conformity bias. While they might score higher on anti-conformity, their learning might be more individualistic than group-oriented. Indeed, research suggests such individualism (the inclination to overlook group associations and treat others as distinct entities rather than group members) is a subcategory of marginalization (Bourhis et al., 1997). Alternatively, marginalized individuals might be conformists, but align with groups defined by factors other than ethnicity, which is the typical dimension examined in acculturation studies (Kunst & Sam, 2013a; Verkuyten et al., 2019).

In societies leaning toward individualism as an ideology or promoting colorblindness, a strategy that emphasizes the development of individual strengths could yield some payoffs. Yet, colorblindness, an ideology that ostensibly proposes treating people without regard to cultural differences, often disadvantages minority groups due to unaddressed systemic racism and inequality (Dovidio et al., 2016). Therefore, from a payoff perspective, marginalization might entail higher costs for minority-group members due to ethnic discrimination, compared with majority-group members. Nevertheless, an individualistic marginalization strategy might be adopted by individuals who have faced discrimination from their ethnic group (e.g., for not being prototypical enough of the in-group), coupled with rejection from other groups (e.g., for being too different). Similarly, regarding prestige bias, marginalized individuals might learn from high-prestige individuals but attribute their success to personal traits and skills rather than cultural backgrounds, thus limiting cultural learning. As such, they would likely emulate traits of role models they perceive individually rather than collectively.

Finally, like other acculturation strategies, marginalization might also partially stem from horizontal and vertical transmission. If parents hold an individualistic or colorblind marginalization preference, this might influence their children to partially adopt this acculturation strategy themselves. Alternatively, these parents might have experienced exclusion from their own and other groups and wish to give their children an alternative perspective that spares them from these experiences.

## Population-Level Consequences of Acculturation Strategies

The cultural evolutionary framework we have built up so far comprises mechanisms of cultural transmission (conformity, prestige bias etc.), which contribute to acculturation strategies at the individual level determined by whether these mechanisms cause individuals to adopt or reject minority and majority cultural elements. The resulting differential adoption of these strategies by different segments of society in turn generates cultural evolutionary equilibria at the level of the society. These equilibria will be reflected in specific distributions of acculturation strategy frequencies

A cultural evolution framework is particularly useful for thinking about how such individual-level interactions and strategies scale up to generate population-level patterns of cultural change and diversity. This utility naturally comes with an evolutionary framework: biologists do the same when they think about how individual-level interactions (e.g., mating, breeding, feeding) scale up to the population-level to generate adaptive radiations, speciation, extinction, and so on (Hartl et al., 1997). And just as in evolutionary biology, cultural evolution researchers typically do this using formal mathematical or agent-based models. This approach is valuable because translating from multiple individual interactions and the strategies they resemble to the population level involves complex dynamics that the unaided human mind finds particularly difficult. In the following sub-sections, we explore the potential consequences of acculturation strategies for patterns of within- and between-group cultural variation; the spread and recombination of beneficial cultural innovations which drive cumulative cultural evolution; cooperation and cultural group selection; and division of labor/specialization. Where possible we discuss existing cultural evolution models of acculturation and how they inform our understanding of each of these phenomena.

### Within-Group Cultural Variation

Differing tendencies to adopt or reject majority and minority cultural traits within a society, as is characterized by different acculturation strategies, will inevitably alter cultural variation within societies. For example, while immigration is a major potential source of increased cultural variation as immigrants bring new languages, religions, customs, foods, and so on into a society, this variation will be less likely to persist if immigrants pursue an assimilation strategy and lose their heritage culture. Integration and separation, on the contrary, seem more likely to maintain cultural variation but in different ways: integration by creating culturally diverse societies and separation by creating segregated societies. Marginalization can perhaps be seen as the ultimate loss of cultural variation, both for the majority and the minority groups.

To test these ideas more formally, Erten et al. (2018) developed a cultural evolution model exploring how different acculturation strategies might or might not maintain a culturally diverse society in which cultural traits common to both immigrants and residents co-exist. Each individual was assumed to vary in two dimensions: the degree to which they interact with culturally dissimilar others, and when they do interact, the tendency to adopt the cultural trait of dissimilar others. There were two traits, one initially held by residents (trait R) and one brought in by a constant influx of immigrants (trait I). Analysis revealed that the two traits were more likely to co-exist (i.e., a culturally diverse society was more likely to persist), when residents and immigrants were more likely to interact, and when residents were less likely to adopt out-group cultural traits than immigrants. Hence, the resident R trait was maintained because residents were more likely to retain it and immigrants more likely to adopt it, while the immigrant I trait persisted due to continual new arrivals into the group.

Although Erten et al.’s (2018) model usefully explores how individual-level social learning and interaction dynamics between majority and immigrant individuals shape cultural diversity, as we advocate here, several limitations prevent its wider application to acculturation more broadly. Perhaps most importantly, the assumption that individuals can only possess one of two cultural traits (R or I) technically precludes a true integration strategy entailing the adoption of both traits simultaneously (or indeed marginalization, involving the lack of either), such that its relation to traditional acculturation strategies is unclear. Indeed, one might view immigrants in their model as pursuing an assimilation strategy of losing trait I and adopting trait R, just at a rate slow enough to allow trait I to persist for some time, which seems an unsatisfying definition of a lastingly “culturally diverse society.” Allowing individuals to possess multiple cultural traits would allow an exploration of the full range of acculturation strategies and also allow for more continuous measures of cultural diversity (e.g., the number of different traits present, rather than one vs. two traits). Nevertheless, Erten et al.’s (2018) broad approach to exploring how acculturation tendencies shape within-society cultural variation is highly promising.

### Between-Group Cultural Variation

Rather than the co-existence of cultural traits within a single society, we can also think about the consequences of acculturation strategies for patterns of cultural variation *between* different societies. In humans, much cultural variation lies between societies (Bell et al., 2009), such as languages, religious beliefs, means of subsistence, and general customs that differ across societies but are relatively uniform within societies. In an explicit model of migration, acculturation, and between-group cultural variation, Mesoudi (2018) modeled how acculturation affects the maintenance of cultural traditions that differ across societies. It is well known from population genetics that migration breaks down group structure: even if two groups are initially genetically entirely distinct, a small amount of migration eventually causes them to become genetically identical. The same logic applies to cultural evolution: a small amount of migration should, in theory, break down any between-group cultural differences between societies. However, while organisms cannot change their genes when they migrate to a new group, people can change their culture, that is, they acculturate. In Mesoudi’s (2018) model, acculturation was assumed to be conformist such that migrants are disproportionately more likely to adopt the majority trait in their new society. This led to either assimilation, when conformity was strong enough to cause migrants to adopt the majority, or separation, when it was not (as in Erten et al. (2018), a weakness of this model is that individuals could possess only one trait at a time, so precluding true integration).

Mesoudi (2018) found that only a small amount of conformist acculturation was needed to maintain between-group cultural traditions at levels that are realistic for human societies (Bell et al., 2009). Fears that immigration inevitably erodes long-standing cultural traditions that characterize different societies are likely unfounded. However, this conclusion crucially depended on the patterns of social interaction in the model. When individuals interacted only with culturally similar others, for example, reflecting a highly segregated society in which immigrants only interact with other immigrants, then acculturation had no effect and migration rapidly broke down between-group traditions. This emphasizes the importance of intercultural contact in maintaining between-group cultural traditions and how within- and between-group cultural variation often trade-off against each other.

One useful aspect of this and other cultural evolution studies (e.g., Bell et al., 2009; Muthukrishna et al., 2020) is the use of a quantitative measure of cultural diversity, cultural *F_ST_*, or cultural “fixation index.” This measure is borrowed from genetics where it is used to quantify the degree to which different populations differ genetically; adapted to a cultural context, cultural *F_ST_* quantifies the degree to which populations differ culturally. Specifically, it measures the ratio of between-group to within-group cultural variation (similar to how an analysis of variance measures the ratio of between versus within-sample variation, resulting in a statistic similarly denoted *F*). When populations are culturally identical and indistinguishable (i.e., all cultural variation lies within not between populations), then *F_ST_* = 0. When populations are entirely culturally distinct (i.e., all cultural variation lies between not within groups), then *F_ST_* = 1. Mesoudi (2018) used cultural *F_ST_* to quantify the effect of migration and conformist acculturation on the stability of between-group cultural variation, comparing the resulting model *F_ST_* values to those found for real-world cultural datasets calculated by Bell et al. (2009). The use of this and other measures from genetics to quantify often informal notions such as “cultural diversity” or “cultural traditions” constitutes another key benefit of a cultural evolution approach.

### Innovation, Recombination, and Cumulative Cultural Evolution

In the previously discussed examples and models, the cultural traits of majority-group members and immigrants are often relatively arbitrary; we would not say that German is intrinsically “better” than Turkish as a language, or individualism “better” than collectivism—they are simply alternative means of communication or self-construal. Sometimes, however, immigrants might bring objectively beneficial knowledge, practices, or technologies into a society. Indeed, historical evidence suggests that regions (e.g., U.S. counties) with higher immigration rates also have higher innovation, as measured using patents, and consequently higher average incomes and employment rates (Akcigit et al., 2017; Nunn et al., 2017). This effect is primarily driven by the increased cultural diversity brought by immigrants (Posch et al., 2023) rather than immigrants bringing any pre-existing economic wealth advantage (indeed, immigrants are often initially at an economic disadvantage). The increased cultural diversity brought by immigrants might constitute either standalone beneficial innovations that are simply adopted wholesale by majority-group members, or traits brought by immigrants that are then recombined with existing technological or social practices already present in the destination country to create something greater than the sum of its parts. Both phenomena facilitate what is known as cumulative cultural evolution, whereby beneficial cultural traits accumulate over time to surpass what any one individual could ever invent or discover alone (Creanza et al., 2017b; Mesoudi & Thornton, 2018). In terms of acculturation strategies, these effects are likely facilitated by a separation or integration strategy among immigrants, plus an integration or assimilation strategy among majority-group members. Rapid assimilation among immigrants will cause any beneficial traits they bring to be lost. Separation might preserve beneficial traits, by integrating or assimilating majority-group members adopting these beneficial traits. However, integration by both immigrants and majority group members is most likely to lead to the recombination of minority and majority traits and the often substantial benefits brought by recombination.

### Division of Labor and Specialization

Sometimes the rapid spread of beneficial cultural traits is, paradoxically, not optimal in the long term. If everyone rapidly converges on the same beneficial cultural trait, then this can prematurely close off other areas of scientific or technological discovery which may yield even greater longer-term benefits (Derex et al., 2018). Similarly, economists since Adam Smith have recognized the value of division-of-labor and specialization, an insight confirmed in cultural evolution models (Henrich & Boyd, 2008). Here, separation on the part of both immigrants and majority-group members is likely to generate such dynamics by maintaining within-society differences in, for example, economic activity, to the overall benefit of society. Historical evidence from different U.S. counties supports this hypothesis. For example, immigration-driven cultural fractionalization into different economic niches (e.g., German Americans specializing in brewing, English Americans in textiles, and Scandinavians in lumber) was a significant predictor of subsequent economic output, compared with both homogeneous low-immigration counties and culturally polarized counties with only two large and competing immigrant minorities (Ager & Brückner, 2013).

### Cooperation and Cultural Group Selection

Migration and acculturation also play key roles in shaping human cooperation. Humans are distinctive in the natural world for our unusually frequent and widespread cooperation among non-kin, which permits us to live in large, relatively cohesive societies. One hypothesis for how this large-scale cooperation evolved is cultural group selection, in which more cohesive and more internally-cooperative societies historically outcompeted less cohesive and less internally cooperative societies (Richerson et al., 2016). While intuitively plausible, theories of group selection are vulnerable to the existence of free riders: individuals who benefit from the cooperativeness of others but do not pay the costs (e.g., paying taxes and fighting in wars) themselves. Within societies, free riders can outcompete cooperators, hence undermining selection for cooperation at the group level. Cultural group selection also requires there to be more cultural variation between groups than within groups; otherwise, groups are not cohesive units that can be subject to selection.

As noted earlier, migration and acculturation are key determinants of both within- and between-group cultural variation and so will crucially influence the evolution of cooperation via cultural group selection. Migration without acculturation breaks down between-group cultural variation (Mesoudi, 2018), preventing groups from acting as cohesive units of selection, and potentially allowing selfish free riders to invade groups of cooperators. This outcome is particularly likely to occur when migration itself is payoff-biased (Boyd & Richerson, 2009; Mesoudi, 2018), meaning that people preferentially move from groups with low average payoffs (e.g., because they contain lots of free riders) to groups with high average payoffs (e.g., because they contain lots of cooperators). Acculturation, however, can act against this and maintain between-group cultural traditions such that cultural group selection can favor more-cooperative societies (Mesoudi, 2018). Acculturation here must take the form of assimilation, that is, immigrants low in cooperation becoming more cooperative when moving to a cooperative society. This process can occur either via conformity, as discussed earlier, or via payoff bias plus some form of punishment of free riders, decreasing its payoffs (Mesoudi, 2018). Large-scale surveys suggest that cooperative cultural norms such as trust in strangers and trust in institutions indeed acculturate in this way, with 2^nd^-generation immigrants from low-trust countries readily adopting the high trust of their new societies (Dinesen & Hooghe, 2010). If conformist or payoff-biased acculturation maintains cooperation within groups, then payoff-biased migration can cause the spread of cooperation across groups, via cultural group selection (Boyd & Richerson, 2009).

Other models of cultural group selection, meanwhile, suggest that acculturation might not necessarily have such benevolent consequences as the spread of cooperative norms. Henriques et al. (2019) show that acculturation itself can drive the spread of intergroup conflict that benefits neither individuals nor societies. Here, more war-like groups that have higher tendencies to engage in conflict are more likely to win those conflicts and spread the war-like tendency to the losing groups via acculturation among the losing groups’ members toward the new majority. This creates a self-perpetuating process, similar to the meiotic drive in genetic evolution, where traits evolve simply because they are good at being transmitted, rather than benefiting the individuals or groups who bear them.

## Future Directions

While few cultural evolution models have attempted to explore the population-level consequences of acculturation (Erten et al., 2018; Henriques et al., 2019; Mesoudi, 2018), these have nevertheless provided valuable insights and promising avenues for further modeling and empirical data collection. Even where formal models do not yet exist, we have explored several areas in which acculturation is likely to shape long-term, population-level cultural change and diversity. Here, we discuss topics and questions that may be profitably addressed in future research (see Table 2).

**Table 2.** Topics and Research Questions for Future Research.

Topics for future research	Potential research questions
● Establishing the relative importance of different mechanisms of cultural transmission (conformity, anti-conformity, payoff bias, prestige bias, vertical transmission) on acculturation	1. Under what circumstances are some mechanisms more influential than others? 2. Are some mechanisms more frequently used for certain cultural domains, or amongst certain categories of people? 3. Do some mechanisms interact (e.g., conformity to high prestige models)?
● Identifying the multiple and sometimes diverging targets of the cultural evolutionary modes of transmission	4. What is the influence of different numerical proportions between groups at different levels (e.g., nation, city, neighborhood) for conformity (e.g., majority minority settings vs. settings in which the historical majority remains largest)? 5. What is the influence of prestigious models at different societal levels? (e.g., entrepreneurs, politicians, community leaders) 6. How do payoffs differ across acculturation domains and the public vs. private spheres?
● Identify how changes in wider societal factors moderate the impact of mechanisms of cultural transmission	7. How do increases in social inequality and/or immigration (e.g., toward superdiversity) or policy shifts influence the transmission of culture? 8. How do these factors affect population-level outcomes?
● The consequences of immigration and acculturation for cohesion versus innovation, relating to the ‘paradox of diversity’	9. Which acculturation strategies at the population level foster cohesion? 10. Which acculturation strategies produce innovation? 11. What equilibria maximize both?

First, it is useful to consider whether learning modes exert primary effects or interact with one another. Conformity, anti-conformity, payoff bias, prestige bias, and vertical transmission may have simultaneous impacts, yet their relative strengths could vary. As previously discussed, in some contexts vertical transmission might primarily contribute to the preservation of one’s ethnic in-group culture, whereas conformity and other biases might facilitate the adoption of out-group cultures. It is also plausible that different cultural evolutionary modes of transmission interact; for instance, conformity may be particularly strong toward groups of high prestige. Yet, the population-level outcomes of such divergent influences remain unclear. Modeling these relationships under various conditions and across different groups could clarify the relative impacts of the modes of transmission and modulating factors highlighted in this review. It would also be valuable to determine whether different types of cultural transmission are utilized in distinct domains. For example, modes of cultural transmission may be employed to varying extents and have different effects in public and private spheres (Arends-Tóth & van de Vijver, 2007).

Future research could also examine the social systems in which individuals reside. The different modes of cultural transmission can function at various levels (e.g., nation, city, neighborhood) in diverse manners. For instance, modeling the outcomes of conformity at different numerical ratios of in-groups to out-groups at these levels would be insightful. While it may be expected that conformity toward one culture is highest when the group constitutes the majority at each level, estimating the relative impact of different conformity targets situated at various levels would be fruitful. It would be insightful to examine the interaction between influences from majority-minority contexts (e.g., at the neighborhood or county level) and contexts where the historical majority group remains numerically predominant (e.g., the national level). Similar questions arise concerning prestige bias, where individuals from different groups may hold varying prestige at different societal levels. In addition, the payoff of adopting or maintaining certain cultural elements is likely to differ across acculturation domains, particularly the public and private spheres as discussed.

Assessing how societal-level factors moderate individual-level modes of cultural transmission and the resulting group and population-level changes is crucial. As explored in this review, social inequality is expected to significantly alter the effects of payoff bias and prestige bias, affecting not only their absolute influence but also the degree to which these biases encourage the adoption of others’ or maintenance of one’s own culture. Importantly, these processes may further perpetuate or shape future societal inequalities. For example, if inequality and discrimination promote assimilation, this could reduce within-group cultural diversity but potentially maintain or even exacerbate discrimination due to racialization or other processes (Dovidio et al., 2016).

While previous research has examined how varying degrees of diversity influence population-level cultural change at different levels of conformity (e.g., Mesoudi, 2018), it would be useful to evaluate the influence of states of super-diversity and how immigration policies and ideologies modify their parameters. For instance, ideologies such as assimilationism might increase the strength of conformity (*f*, see Table 1) toward the majority group, whereas multiculturalism or polyculturalism might encourage simultaneous conformity toward multiple groups. Likewise, under multiculturalism, prestigious individuals in society, particularly those from minority backgrounds, might publicly express their ethnic heritage, potentially leading both minority- and majority-group members to adopt this culture more than under ideologies (e.g., colorblindness) that discourage cultural pride.

Intergroup ideologies may also influence the processes of interest by increasing or decreasing intercultural interaction. Future models and empirical research might explore how different forms of social organization that are often tied to these ideologies affect patterns of social interaction, and in turn the population-level phenomena discussed above. Existing models already incorporate simple forms of interaction patterns, and despite this simplicity, often find dramatic effects. For example, both Erten et al. (2018) and Mesoudi (2018) found that the spread of immigrant-related cultural traits crucially depends on the probabilities of immigrants and majority group members interacting. Further models can extend these simple implementations, exploring how more realistic and diverse forms of social networks (Cantor et al., 2021) interact with acculturation and migration to shape cultural change and diversity.

Finally, there seems to be a broad trade-off related to what Schimmelpfennig et al. (2022) have called the “paradox of diversity.” Cumulative cultural evolution is facilitated by increased cultural diversity: beneficial new inventions, discoveries or practices, are often brought by immigrants as discussed above. Hence, cumulative cultural evolution is favored by acculturation strategies that maintain and increase diversity within societies such as integration and separation. However, increased cultural diversity within societies also often comes with the loss of social cohesion and the breakdown of long-standing cultural traditions (see Dinesen et al., 2020). This loss of cohesion can be detrimental to cooperation within human societies. Hence, cooperation is favored by acculturation strategies that reduce cultural diversity within societies such as assimilation. There is great potential for cultural evolution models to formalize this trade-off and make empirical predictions about how different acculturation strategies might balance this trade-off. Perhaps different cultural traits acculturate at different rates or with different strategies, as was found empirically by Bell (2013) for various Tongan practices and beliefs among Tongan-Americans. Hence cooperation-related traits such as trust in strangers might be subject to assimilation to facilitate cultural group selection, while technological traits such as new computing devices might be subject to integration to facilitate recombination and cumulative cultural evolution. Other traits for which lengthy training or apprenticeship is needed might be subject to separation to facilitate division-of-labor and specialization.

## Constraints on Generality and Citation Statement

Before concluding, we consider constraints on generality (Simons et al., 2017) and limitations of the work cited in this review. The field of acculturation is characterized by ethnic, religious, and gender diversity and emphasis on cross-cultural research, as reflected in the cited studies. These include samples from both Western and non-Western countries and from various minority and majority groups, thereby contributing to a broad and inclusive understanding of acculturation processes. However, while many of the cited authors are themselves from non-Western minority groups, a significant portion are Caucasian and from Western countries. Moreover, many of the theoretical models we rely on were developed within Western contexts. While we have sought to include work from a broad range of scholars, we acknowledge that the diversity of authorship is skewed.

Our framework is also limited in that it partly builds on an essentialist account of minority and majority cultures that has been highlighted as a main limitation of dominant acculturation models (Doucerain, 2019; Doucerain et al., 2013). Moreover, for the sake of simplicity, we often consider immigrant and minority group cultures combined. It is crucial to recognize the extensive heterogeneity within these categories. They encompass a wide array of groups whose cultures can be entirely distinct or partially overlapping, each bearing unique historical and socio-political backdrops and sometimes competing political interests (Hindriks et al., 2017) that likely shape the processes we outlined (see Verkuyten, 2018).

Evolutionary models typically also assume one of two discrete cultural traits, whereas many cultural dimensions of interest to acculturation researchers are continuous, not discrete. Cultures, by their nature, are fluid and constantly changing. As such, the assumption of distinct cultural groups is a simplification. Continuous traits are more amenable, for example, to blending together the values of multiple social sources in a way that discrete traits are not. Social interaction in many evolutionary models is also simplified, assuming either random interactions or some probability of interacting with similar others. Real people inhabit overlapping social networks and exhibit overlapping identities beyond simply “immigrant” and “non-immigrant.” Phenomena such as chain migration, remittances, and government policy add further complications.

Our focus has been on acculturation strategies and orientations in a general sense. However, acculturation occurs across specific societal spheres and life domains. Consequently, while individuals undergoing acculturation may predominantly fall into a particular category, their acculturation patterns are more nuanced and can vary significantly across different domains (Doucerain, 2019; Navas et al., 2005; Rojas et al., 2014; Rudmin, 2003). Also, there is variation even within these acculturation strategies. While some individuals may embody a certain strategy quite typically, others might find themselves straddling several strategies.

In one sense, the aforementioned simplifications are necessary: a model that is as complex as reality is of no use because one might as well study reality; instead, a model’s usefulness (just like an experiment’s) lies in its simplification of a complex phenomenon so that one can better understand and systematically manipulate its general components. Yet, while necessary for model construction, such simplifications limit the generalizability of our theorizing and require extensive testing to establish ecological validity.

When integrating perspectives from acculturation and cultural evolution, we do not aim to reduce complex cultural processes to biological determinism, but rather to explore how these two perspectives could complement and inform each other. Although we have described how the proposed processes are influenced by many individual, group, and contextual factors, our analysis cannot fully encompass all these factors, nor do we claim them to be universally applicable. Cultural change is a complex process, shaped by many factors beyond those discussed here (see, e.g., Kitayama & Uskul, 2011; Uskul & Oishi, 2020; Varnum & Grossmann, 2017). We examined five pivotal processes core to the cultural evolution literature, each substantiated through meticulous research exploring their respective impacts on cultural evolution. This body of literature, akin to other fields, perpetually evolves and is anticipated to encompass additional processes in the future. Take, for example, identity signaling, which has predominantly been used to elucidate consumer behavior and, to a degree, group dynamics (J. Berger, 2008; J. Berger & Heath, 2008). Identity signaling is a promising contender for an independent mechanism of cultural transmission, given the reliance of individuals on group coalitions, which in turn can drive cultural conservation (Boyer et al., 2015; Tooby & Cosmides, 2010). Future research should model its influence on population-level outcomes and explore its potential interactions with strategies like payoff or prestige bias, as suggested earlier.

Numerous interpersonal processes, capable of instigating enduring cultural shifts, have yet to be integrated into the principal framework of cultural evolution. For instance, the dynamic theory of social impact (Latané, 1996) illustrates how individuals impact one another through a cyclical process of influence, negotiation, and consolidation (also see Tindale & Kameda, 2017). This process can forge more or less stable cultural clusters in identities, values, and practices, which can be transmitted across time and space. While interpersonal processes are inherent in some of the modes of cultural transmission (e.g., conformity) and moderated by factors included in our review (e.g., residential segregation), future research might further incorporate this interpersonal perspective. Importantly, the five forms of cultural transmission central to this review are unlikely to be independent of one another. For instance, conformity is not merely a numerical function but intersects with other processes. The prestige of the conformity target and the perceived payoff of adopting the trait constitute the “strength of evidence” that can increase conformity as per the theoretical framework of the burden of social proof (MacCoun, 2012, 2015).

Finally, while we have discussed the influence of characteristics of intergroup relations (e.g., social inequality, spatial segregation, and ideologies), it might be argued that our model presupposes at least a semblance of peaceful relations between groups, reminiscent of many, but not all, ethnically diverse societies globally. Persuasive research has shown how intergroup conflict can be a driver of acculturation, as the victor’s culture is often imposed upon or emulated by the defeated group (Henriques et al., 2019). Future research might introduce intergroup conflict as an additional variable in our model, exerting its own influence on acculturation or moderating those of other processes, such as conformity, payoff, or prestige biases.

## Conclusion

This review has aimed to integrate perspectives from cultural evolution with those of psychological acculturation research. We contend that cultural evolutionary mechanisms—such as conformity and anti-conformity, prestige bias, payoff bias, and vertical transmission—are instrumental in comprehending how minority- and majority-group members retain their culture and/or adopt the culture of other groups. The efficacy of these mechanisms is modulated by contextual and individual factors, collectively accounting for individuals’ and populations’ overall acculturation strategies, which form cultural evolutionary equilibria. The frequency of acculturation strategies in turn has major consequences for population-level phenomena such as the balance between within-society cultural diversity and between-society cultural differences, cumulative cultural evolution and the spread of beneficial technologies, cooperation and cultural group selection, and division of labor. We emphasize that formal cultural evolution modeling, informed by rigorous empirical acculturation research, can illuminate broader patterns of cultural diversity and change, and clarify the “adaptiveness” of different cultural orientations and strategies. By bridging these two fields, we not only enhance our theoretical understanding of acculturation processes but also offer novel tools and perspectives to better address the real-world challenges of ethnically diverse societies.

## Notes

1. We recognize that numerous factors, including familial background, have played a pivotal role in shaping Musk’s success. Despite this, we have selected him as an exemplar given his extensive following and high-profile stature, making him a suitable example for prestige bias.
